# Bile promotes *Lactobacillus johnsonii* N6.2 extracellular vesicle production with conserved immunomodulatory properties

**DOI:** 10.1038/s41598-024-62843-0

**Published:** 2024-05-28

**Authors:** Reagan E. Beliakoff, Claudio F. Gonzalez, Graciela L. Lorca

**Affiliations:** Department of Microbiology and Cell Science, Genetics Institute, Institute of Food and Agricultural Sciences, University of Florida, Gainesville, FL USA

**Keywords:** Bacteria, Bacteriophages, Cellular microbiology

## Abstract

Recently, *Lactobacillus johnsonii* N6.2-derived extracellular vesicles (EVs) were shown to reduce apoptosis in human beta cell lines and stimulate insulin secretion in human islets. Our goal was to identify a physiologically relevant environmental condition that induces a hypervesiculation phenotype in *L. johnsonii* N6.2 and to evaluate if transcriptional changes are involved in this process. Culturing this strain in the presence of 0.2% bovine bile, which mimics a stressor encountered by the bacterium in the small intestine, resulted in approximately a 100-fold increase in EVs relative to cells grown in media without bile. Whole transcriptome analysis of cells grown with bile revealed upregulation of several peptidoglycan hydrolases as well as several genes involved in fatty acid utilization. These results suggest that the hypervesiculation phenotype may be the result of increased cell wall turnover combined with increased accumulation of phospholipids, in agreement with our previous proteomic and lipidomics results. Additionally, EVs isolated from *L. johnsonii* N6.2 grown in presence of bile maintained their immunomodulatory properties in host-derived βlox5 pancreatic and THP-1 macrophage cell lines. Our findings suggest that in *L. johnsonii* N6.2 vesiculogenesis is significantly impacted by the expression of cell wall modifying enzymes and proteins utilized for exogenous fatty acid uptake that are regulated at the transcriptional level. Furthermore, this data suggests that vesiculogenesis could be stimulated in vivo using small molecules thereby maximizing the beneficial interactions between bacteria and their hosts.

## Introduction

The production of extracellular vesicles (EVs) is ubiquitous among all domains of life. EVs are nanosized lipid enclosed particles that carry various biomolecules and exist independently from the cell they originate from until lysis. The functions of EVs are diverse and depend on several factors, including the parent cell that they are derived from and their mechanism of formation. In eukaryotes, EVs called exosomes are formed from late endocytic membranes, whereas microvesicles are formed by budding of the plasma membrane^1^. Exosomes have been shown to transport specific cargos that are unique to each cell type and serve as crucial agents for intercellular communication. These cargos can interact with target cells through surface components, facilitate the transfer of activated receptors, and even induce epigenetic modifications in recipient cells^2^. Similarly, both gram-positive and gram-negative bacteria have been shown to produce EVs through different mechanisms of biogenesis. Recent reports have shown that bacterial EVs can serve as important effector molecules in the context of host:microbe interactions. These interactions can have a detrimental effect on the host when they carry pathogenicity determinants^3,4^, or a beneficial effect on the host when the EVs are derived from probiotic bacteria^5–7^. Consequently, bacterial EVs play crucial roles in interkingdom communication which are dependent on both the species of EV origin and the host cell type they are interacting with.

Structural and chemical differences in the cell envelope of gram-negative and gram-positive bacteria result in unique mechanisms of EV biogenesis. Gram-negative bacteria can generate outer membrane vesicles (OMVs) by budding from the outer membrane, which may transport periplasmic components. Alternatively, cell lysis can lead to the formation of outer-inner membrane vesicles (OIMVs) containing two lipid bilayers that encapsulate cytoplasmic components^8^. In contrast, mechanisms of EV biogenesis in gram-positive bacteria have been less studied due to the previously held belief that the thick peptidoglycan cell wall would render them unable to release EVs. It has been proposed that gram-positive bacteria release EVs by budding at the cytoplasmic membrane driven by turgor pressure at phospholipid enriched patches. The budding EVs are then released through pores in the peptidoglycan generated by peptidoglycan hydrolase (PGH) activity^9^. This hypothesis has been largely supported by -omics studies involving comparative analyses of the lipid and protein compositions of bacterial EVs with those of their parent cell membrane or whole cell fractions. Our group and others have shown that EVs from *Lactobacillus* species have enriched concentrations of PGHs^6,7,10^ in proteomic analyses while an enrichment in phospholipids was observed in the EVs by untargeted lipidomics^11,12^.

In bacteria, multiple stress conditions have been shown to promote hypervesiculation (Recently reviewed^13^). For example, oxidative stress is correlated with changes in EV production by *Campylobacter jejuni*, *Pseudomonas aeruginosa*, and *Streptococcus mutans*^14–16^. Multiple antibiotics have been shown to induce EV production, including cell wall synthesis disruptors, RNA and protein synthesis inhibitors, and genotoxic agents such as mitomycin C^17–19^. In *Lacticaseibacillus casei* BL23, mitomycin C was shown to trigger prophage lytic cycle resulting in increased EV formation^20^. Additional studies have implicated prophage induction as a driver of vesiculogenesis in several gram-positive bacteria. This effect has been attributed to the induction of prophage-encoded lysins that are utilized during the lytic cycle to degrade peptidoglycan and facilitate the release of phage particles^20–22^. Additional cultivation conditions, including alkaline and static growth, were shown to increase EV formation by *Lacticaseibacillus casei* and *Lactiplantibacillus plantarum*, respectively^23^.

With the growing body of evidence indicating that EVs may be involved in interkingdom signal transduction, there is a critical need to understand the conditions that influence their biogenesis and the potential impact of these conditions on their cargo composition. Our lab studies the responses triggered in the host by the probiotic bacterium *Lactobacillus johnsonii* N6.2. After our initial observation that the administration of *L. johnsonii* N6.2 can mitigate the onset of type 1 diabetes (T1D) in BioBreeding Diabetes-Prone rats, we have shown that the pathways affected by this microorganism are context dependent, with local and systemic effects being observed^12,24–27^. *L. johnsonii* N6.2 modulates many immune and metabolic processes. Among them are the inhibition of the activity and expression of the indoleamine 2,3-dioxygenase in ileum and brain tissues^25^, inhibition of inflammasome assembly in the liver^27^, and induction of a Th17 bias in mesenteric lymphatic nodes^26^. The observed systemic effects of *L. johnsonii* N6.2 administration suggested that this bacterium produces bioactive components that interact with host cells at distal locations. Subsequent analyses revealed that the EVs derived from this organism may be mediating the observed effects^5,12^. We observed that *L. johnsonii* N6.2 produces EVs with differentially enriched proteomic and lipidomic profiles when compared to the cell membrane^12^. Enriched proteins in the EVs allowed for the development of EV biomarkers, which were used to evaluate IgA and IgG responses to EVs in healthy human subjects. Plasma from individuals who were administered *L. johnsonii* N6.2 had a significant increase in IgA and IgG specific to the EVs compared to individuals administered the placebo. These EVs were also shown to induce an M2b tolerogenic phenotype in human macrophage cell lines, reduced apoptosis of human pancreatic beta cells, and modulated glucose homeostasis by increasing insulin secretion in human islets^5^.

Based on these findings, we hypothesize that interactions between *L. johnsonii* N6.2 and host cells can be mediated by the production and availability of EVs. In this report, we identified a physiologically relevant cultivation condition that promotes hypervesiculation in *L. johnsonii* N6.2. Using this condition, the changes in gene expression correlated with an increase in EV production were evaluated. Furthermore, the impact of hypervesiculation inducing conditions on host responses was evaluated by measuring the stimulation of an RNA sensing response in human βlox5 pancreatic beta cells as well as innate immune responses in human THP-1 macrophage cell lines.

## Results

### Bile increases the size and quantity of *L. johnsonii* N6.2 derived EVs

**Figure 1:**
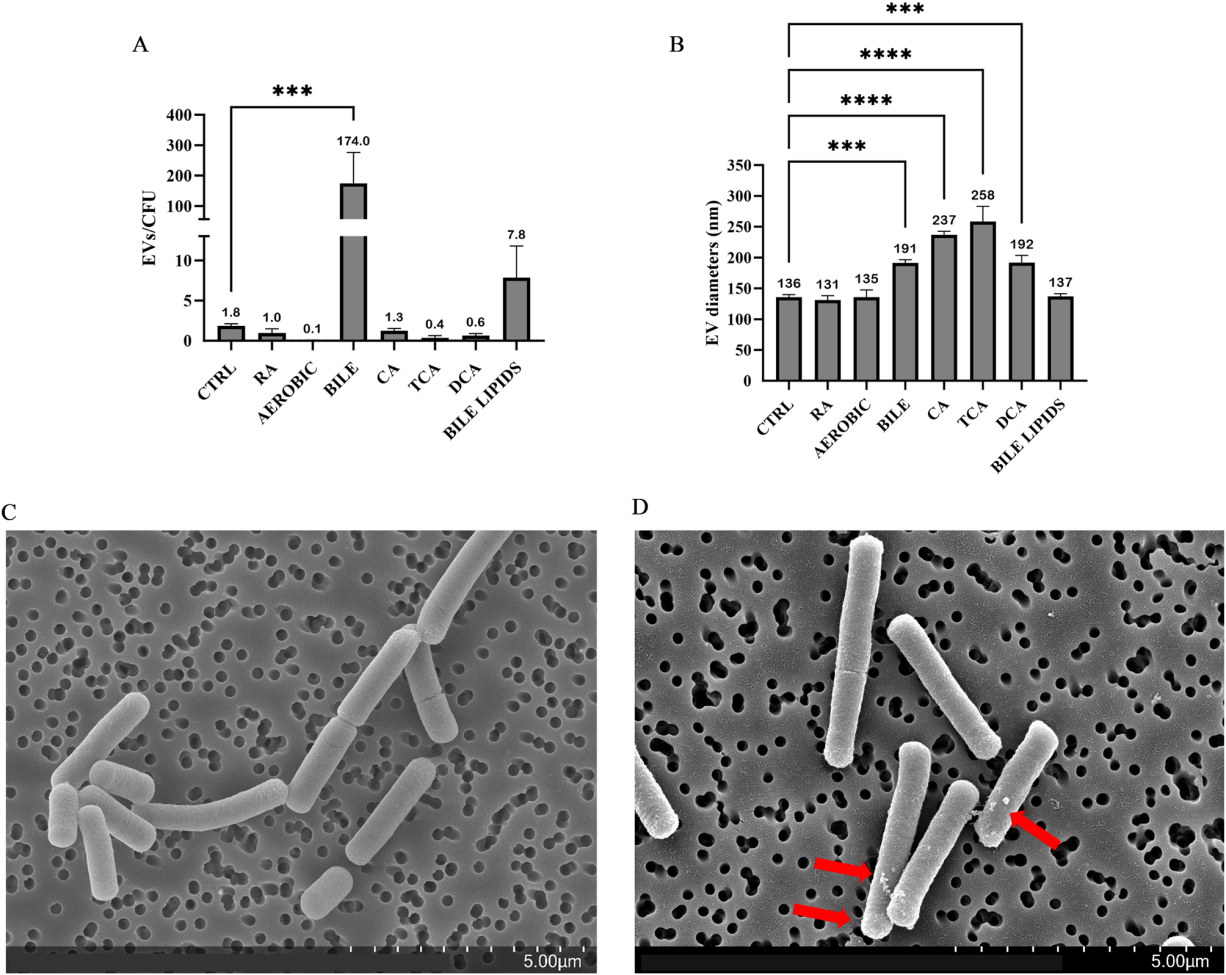
Bile alters the vesiculation pattern and morphology of *L. johnsonii* N6.2. (**A**) EVs/CFU were calculated for *L. johnsonii* N6.2 cultivated statically in vdMRS (CTRL) and with the following modifications: 100 µM rosmarinic acid (RA), shaking at 75 rpms (AER), 0.2% bovine bile (BIL), 2.5 mM cholic acid (CA), 12.5 mM taurocholic acid (TCA), and 7.5 mM deoxycholic acid (DCA), and 0.02% bile lipids (BIL-LIP). (**B**) Average diameters of EVs recovered from each culture condition as determined by NTA. EV isolation was performed in biological triplicates, annotations show the mean of the three biological replicates, and error bars represent standard deviation. Significance was determined using a one way ANOVA with post-hoc Tukey HSD. *p < 0.05, **p < 0.01, ***p < 0.001, ****p < 0.0001. (**C**, **D**) Representative SEM images of late exponential phase *L. johnsonii* N6.2 cultures grown in (**C**) vdMRS in the absence of 0.2% bovine bile, or (**D**) vdMRS in the presence of 0.2% bovine bile.

The impact of environmental conditions, including oxidative stress, dietary phenolic compounds, and bile encountered during gastrointestinal transit, on *L. johnsonii* N6.2 EV biogenesis was analyzed. Oxidative stress was selected because it has been identified as an inducer of EV production in other bacterial species^14,28^. On the contrary, rosmarinic acid was tested as the inverse because it is a dietary phytophenol that was previously shown to rescue *L. johnsonii* N6.2 growth under conditions of oxidative stress^29^. Bile was selected as a physiologically relevant stress condition encountered by the bacterium as it passes through the small intestine. To evaluate EV production, *L. johnsonii* N6.2 was grown in vesicle-depleted MRS (vdMRS) aerobically (75 rpm) to induce oxidative stress, or under static conditions in the presence of rosmarinic acid (100 uM), or bovine bile (0.2%). These concentrations were selected based on evaluations of growth curves performed with increasing concentrations of each compound (Supplementary Fig. 1). We aimed to test concentrations that had a negative but subinhibitory effect on growth in order to capture measurable differences in EV production caused by external stressors affecting bacterial physiology. *L. johnsonii* N6.2 grown under static conditions in vdMRS resulted in 1.8 ± 0.3 EVs/CFU with an average diameter of 136 ± 4 nm. Growth of *L. johnsonii* N6.2 under aerobic conditions or rosmarinic acid did not result in a significant difference in the number of EVs/CFU relative to the control (Fig. 1A). Interestingly, growth in the presence of bile resulted in 174 ± 102 EVs/CFU, almost a 100-fold increase compared to the control (Fig. 1A). Additionally, bile promoted a significant (p < 0.05) increase in the average diameters of the EVs from 136 ± 4 nm to 191 ± 5.5 nm (Fig. 1B).

The effects of bile on the morphology of *L. johnsonii* N6.2 were investigated using scanning electron microscopy (SEM). Cells grown in 0.2% bile showed a rougher surface, particularly towards the poles of the cells, when compared to cells grown in the absence of bile (Fig. 1C,D).

Next, we evaluated whether the hypervesiculation phenotype may be induced by pure bile components. We hypothesized that the detergent-like properties bile acids could affect the fluidity of the *L. johnsonii* N6.2 membrane leading to increased EV budding and release^30^. Growth curves were performed in presence of primary bile acids (cholic acid, CA; taurocholic acid, TCA; glycocholic acid, GCA) and secondary bile acids (deoxycholic acid, DCA; taurodeoxycholic acid, TDCA; and glycodeoxycholic acid, GDCA). Each of these bile acids were tested using a range of physiologically relevant concentrations to identify conditions with an inhibitory but non-lethal effect on growth (Supplementary Fig. 2). Concentrations up to 25 mM of TCA and GCA did not affect growth of *L. johnsonii* N6.2. TDCA and GDCA showed growth inhibitory effects at 15 mM and 2.5 mM, respectively. Deconjugated bile acids such as CA and DCA showed different degrees of toxicity, with CA being more inhibitory that DCA (5 mM and 7.5 mM, respectively). Based on these results, three representative bile acids were selected to test for their effects on EV production. CA (2.5 mM) and DCA (7.5 mM) were selected as a primary and secondary deconjugated bile acids (respectively), while TCA (12.5 mM) was selected as a conjugated bile acid without an inhibitory effect on *L. johnsonii* N6.2 growth. No significant differences in EVs/CFU were found when *L. johnsonii* N6.2 was cultivated with each of these three bile acids at the selected concentrations (Fig. 1A). However, the average diameter of EVs recovered from each group were significantly larger than the control (p < 0.05), similar to the results found with whole bile solution (Fig. 1B). These results suggest that individual bile acids are not solely responsible for promoting the bile-induced hypervesiculation phenotype.

Another major component of bile is lipids^30^. To test whether these lipid components may be responsible for promoting EV biogenesis, total lipids were extracted from vesicle-depleted bile. The total yield was approximately 100 mg dried lipids per gram of lyophilized bile, or a 10% yield. Based on this yield, the effect of 0.02% bile lipids was evaluated, so that the lipid concentration was equivalent to the crude bile solution. The addition of purified lipids to the media did not affect the growth of *L. johnsonii* N6.2 (data not shown) nor the size of the purified EVs (Fig. 1B). However, an increase in the number of EVs/CFU from 1.8 ± 0.3 to 7.8 ± 4 EVs/CFU was observed although this change was not statistically significant (Fig. 1A). These results indicate that lipids from bile are likely a contributing factor to the hypervesiculation phenotype induced by bile.

Lastly, we attempted to investigate the impact of bilirubin, a component of bile that is produced as the end product of heme metabolism, on the hypervesiculation phenotype. However, bilirubin is only soluble in aqueous solutions at low concentrations, precluding the evaluation of physiologically relevant concentrations equivalent to the whole bile. Furthermore, the ultracentrifugation step used for EV isolation resulted in the profuse precipitation of bilirubin, masking the EV production from *L. johnsonii* N6.2.

### Bile induces significant chromosome-localized transcriptional changes in *L. johnsonii* N6.2

**Figure 2:**
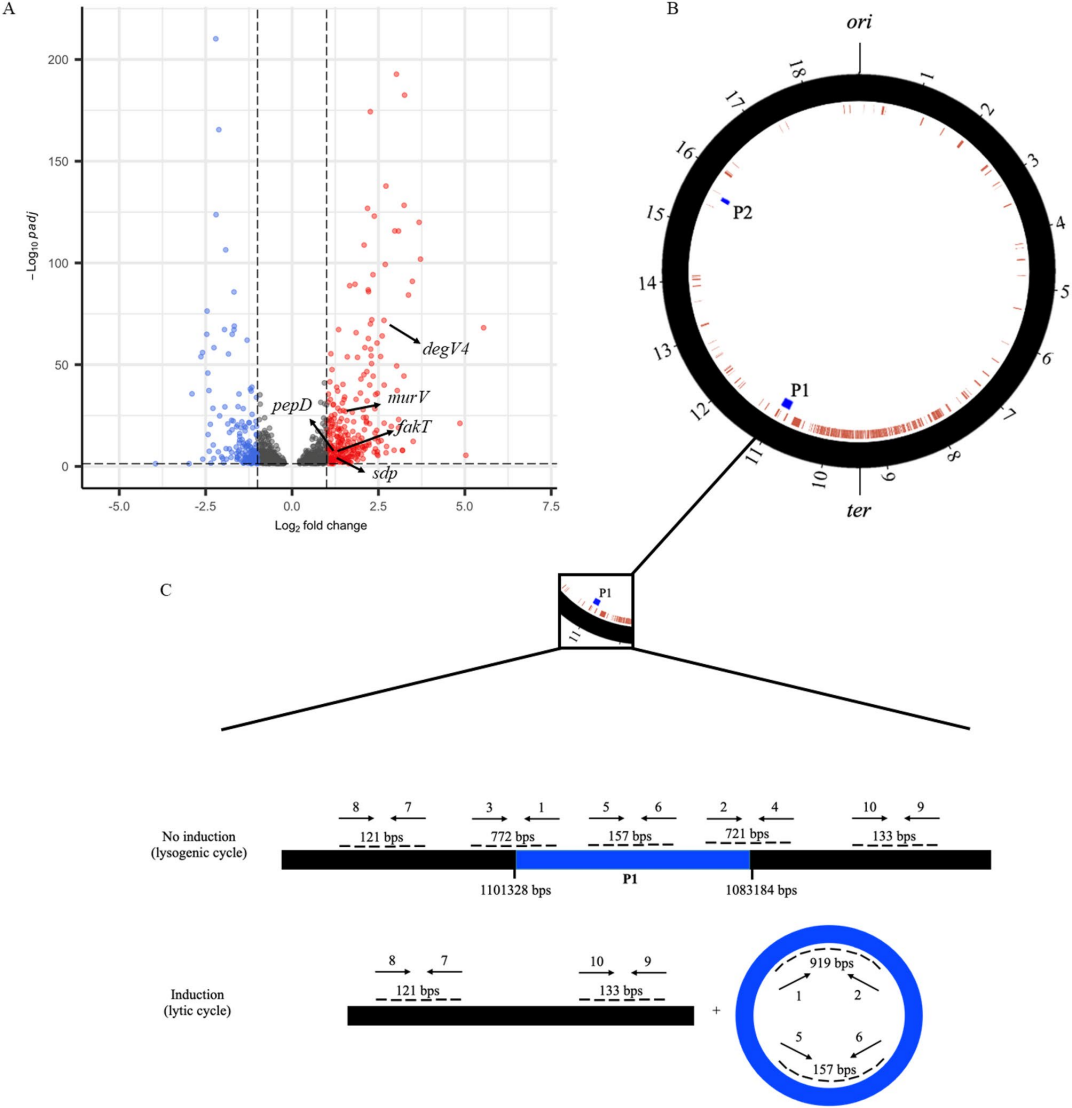
Genes induced by bile are localized to a specific 300 kb region of the *L. johnsonii* N6.2 genome. (**A**) Volcano plot showing significantly upregulated genes (red) and downregulated genes (blue) when *L. johnsonii* N6.2 is grown with 0.2% bovine bile. (**B**) Genomic locations of incomplete prophage regions and genes induced when *L. johnsonii* N6.2 is cultivated with bile. Black circle represents *L. johnsonii* N6.2 chromosome, red arcs represent genes induced during growth with bile log2 Fold Change > 1, blue arcs represent incomplete prophage regions predicted by PHASTER. Tick marks are 1 = 100 kb. (**C**) Schematic representation of the primer locations around the putative prophage 1 (P1). Figure not to scale.

To elucidate genes that may be contributing to EV biogenesis, we performed RNA-seq on *L. johnsonii* N6.2 cells grown in 0.2% bile. When compared to the control, cells grown in 0.2% bile showed a significant change (padj < 0.05) in 967 transcripts. Of these transcripts, 269 were upregulated with a log_2_ fold change > 1 and 161 were downregulated with a log_2_ fold change < − 1 (Fig. 2A, Supplementary Table 1). Despite the large number of genes that were significantly differentially expressed, there were no GO terms or KEGG pathways that were significantly induced by bile.

Among the 25 genes significantly downregulated in presence of bile (log_2_ fold change < − 2), eight genes are annotated as putative transporters. Bile stress has been shown to increase bacterial membrane permeability^31^, so reduction in the expression of various transporters may be a stress response to reduce internalization of harmful bile components. Two of these downregulated transporters were identified as potential bile salt transporters using the transporters classification database (TCDB)^32^. While many enteric bacteria employ bile salt efflux as a mechanism to resist bile stress, *Lactobacillus johnsonii* 100–100 is noteworthy for encoding two bile salt importers CbsT1 (AAC34379.2) and CbsT2 (AAC34380.1)^33^. These importers are gene repeats encoded in tandem with a bile salts hydrolase (BSH) that demonstrated activity for taurocholic acid^33^. CbsT1 shares 99% sequence identity with T285_RS00305 (log2FC = − 2.26) while CbsT2 shares 99% sequence identity with T285_RS00300 (log2FC = − 2.21). CbsT2 has been characterized as a taurocholate:cholate antiporter responsible for uptake of taurocholic acid for deconjugation by the downstream BSH, then subsequent release of the resulting cholic acid^34^. The *bsh* gene (T285_RS00295) directly downstream of these transporters was also significantly repressed by bile (log2FC = − 1.75), and the protein product shares high sequence homology (98%) with the BSH (AAC34381.1) from *L. johnsonii* 100–100.

**Figure 3:**
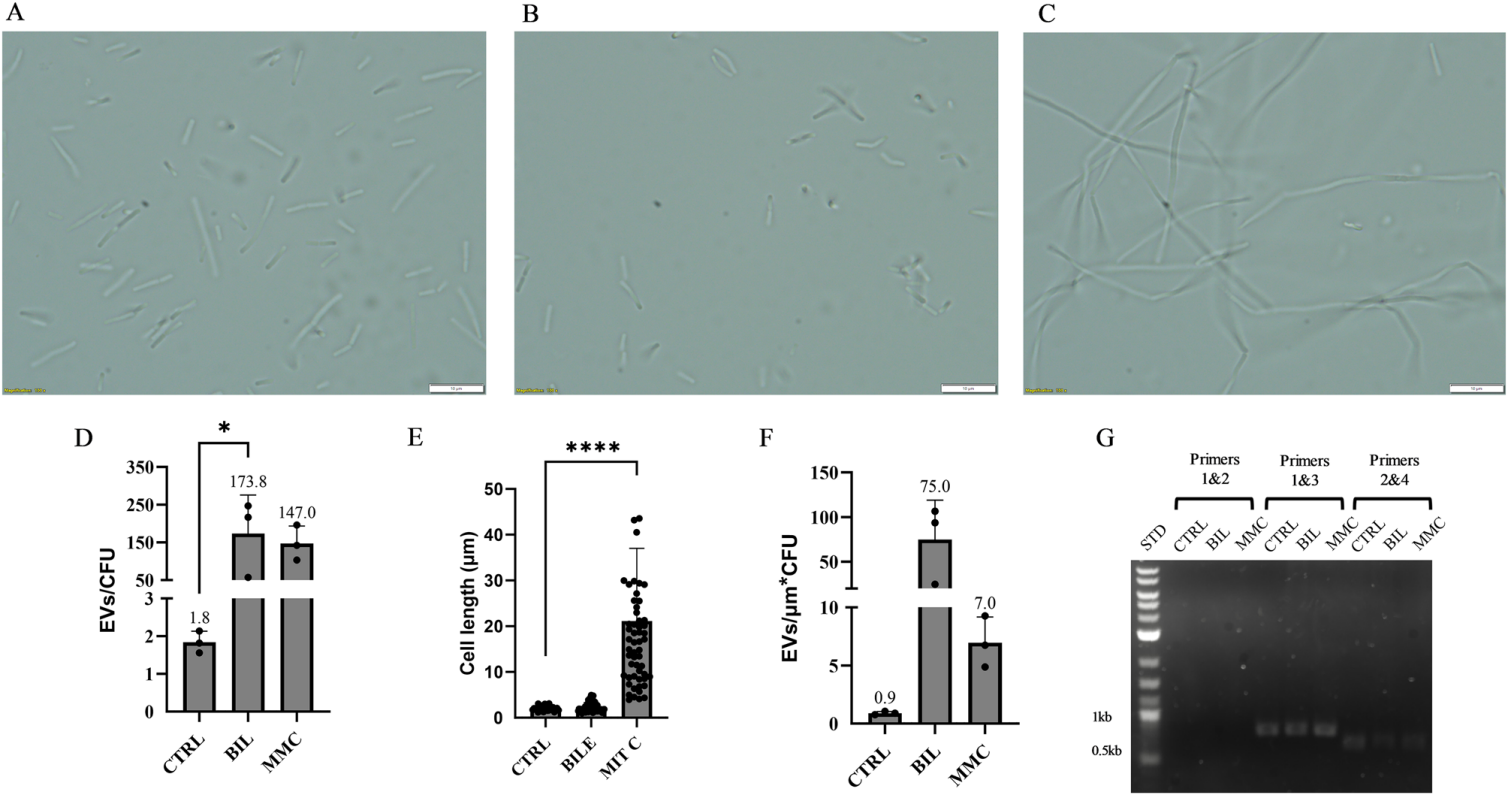
The increase in EV/CFU triggered by MMC is attributed to an increase in cell length rather than induction of a prophage. (**A**–**C**) Bright field microscope images 100X of *L. johnsonii* N6.2 grown to late exponential phase in vdMRS media, vdMRS media with 0.2% bovine bile, and vdMRS media with 116.7 nM mitomycin C (MMC), respectively. (**D**) EVs/CFU were calculated for *L. johnsonii* N6.2 cultivated statically in vdMRS (CTRL) and with the following modifications: 0.2% bovine bile (BIL), 116.7 nM MMC. EV isolation was performed in biological triplicates annotations show the mean of the three biological replicates, and error bars represent standard deviation. (**E**) Average cell lengths of *L. johnsonii* N6.2 cells from CTRL, BIL, and MMC cultures. Annotations show the mean length of 30 cells, and error bars represent standard deviation. (**F**) EVs/CFU divided by the average cell length of each respective group. Significance was determined using a one way ANOVA with post-hoc Tukey HSD. *p < 0.05, **p < 0.01, ***p < 0.001, ****p < 0.0001. (**G**) PCR reactions to test for the prophage P1 induction using DNA extracted from *L. johnsonii* N6.2 cells from CTRL, BIL, and MMC cultures. Primer numbers correspond with Table 2.

Interestingly, of the 269 genes induced with a log_2_ fold change > 1, 184 genes (68%) were localized to a specific 300 kb region of the *L. johnsonii* N6.2 genome (Fig. 2B). This 300 kb region accounts for only 16% of the total genome sequence. The marked chromosome localization of genes induced by bile prompted us to investigate the presence of a prophage in this region that, upon stimulation by bile, could be entering the lytic cycle thereby promoting transcriptional changes in the area. This would also explain the increase in EVs produced during growth with bile, as prophage induction has been implicated as a driver of EV release in *Bacillus subitlis*, *Lactococcus lactis*, and *Lacticaseibacillus casei*^20–22^. In silico prophage prediction of the *Lactobacillus johnsonii* N6.2 genome (NC_022909.1) using the PHASTER software^35^ identified two incomplete prophages (P1 and P2) (Fig. 2B). P1 is located at 1,083,184–1,101,328 bps and P2 at 1,555,314–1,564,170 bps, and both regions lack repressor genes, holin-lysin systems, and many structural genes required to generate full phage particles. P1 is the larger of the two regions and is located directly downstream of the 300 kb region of the genome that was highly induced by bile. To evaluate if a prophage may be responsible for the hypervesiculation phenotype, the effect of mitomycin C (MMC) on EV production was assessed. This genotoxic agent is commonly used to induce the production of temperate phages from lysogenic strains^36^. Remarkably, when *L. johnsonii* N6.2 was grown in presence of a sublethal concentration of MMC (116.7 nM, Supplementary Fig. 3), there was a significant increase in the number of EVs/CFU (147 ± 47 EVs/CFU) despite the apparent lack of an inducible prophage that could be contributing to this phenotype (Fig. 3). To examine whether the region 1 incomplete prophage sequence was excised during growth with MMC, an inverted PCR reaction was conducted using primers 1 and 2, running out of either side of the predicted prophage sequence (Figs. 2C, 3). It was expected that during prophage induction this region would be excised and circularized, resulting in a 919 bp PCR product. As PCR controls, primers located outside of the area (3 and 4) were utilized. PCR reactions were performed using DNA extracted from *L. jonsonii* N6.2 grown in absence or presence of 0.2% bile or 116.7 nM MMC. It was found that primers 1 + 2 did not produced a visible DNA band, confirming that the predicted incomplete prophage is not induced (Fig. 3G). In agreement with this result, the use of the other primer combinations outside of the area resulted in the expected DNA fragments.

The inverted PCR results were further confirmed by quantifying the enrichment of DNA in the prophage region. Amplification of sequences within the incomplete prophage sequence revealed no enrichment in the DNA of *L. johnsonii* N6.2 grown in bile or MMC when normalized to a sequence outside of the incomplete prophage (Supplementary Fig. 4A,B). These results confirmed that induction of a prophage is not involved in promoting EV biogenesis in *L. johnsonii* N6.2. These findings agree with a previous report demonstrating that *L. johnsonii* NCC 533 contains genomic sequences derived from prophages, but these are not inducible^37^.

Next we examined whether changes in cell morphology resulting from growth in presence of MMC could skew the EV quantifications observed. *L. johnsonii* N6.2 cells grown in MMC were approximately 10 times longer than cells grown in standard vdMRS or in the presence of bile (Fig. 3A–C,E). Based on this observation, the EV counts were normalized to both the number of viable cells in the culture and average length of these cells. The EVs/CFU measurements previously determined for each condition were divided by the average length in micrometers of the bacterial cells (Fig. 3F). This estimation revealed that there was no significant difference in the number of vesicles produced by *L. johnsonii* N6.2 grown with MMC when both length and viable cells were accounted for. These results highlight the importance of examining cell morphology combined with viability and vesicle quantification during the analyses of vesiculation patterns in bacteria.

## Transcriptome analysis of *L. johnsonii* N6.2 grown in 0.2% bile reveals induction in the expression of peptidoglycan hydrolases and fatty acid utilization genes

The analyses of the transcriptome data showed that the expression of 19 genes was highly induced (log_2_ fold change > 3) in the presence of bile (Supplementary Table 1). Among these highly induced genes are mevalonate biosynthesis genes (T285_RS04565, T285_RS04560, T285_RS04570, T285_RS04555) and genes involved in DNA modification and replication (T285_RS04630, T285_RS04575, T285_RS04740, T285_RS04575, T285_RS04580) (Supplementary Table 1). The mevalonate pathway is not well characterized in *Lactobacillus*, but in *Staphylococcus aureus*, mevalonate was shown to be a precursor for peptidoglycan synthesis^38^. We hypothesize that growth of *L. johnnsonii* N6.2 in presence of bile may disrupt cell wall homeostasis, and this defect maybe compensated for by the upregulation of genes required for peptidoglycan synthesis. These results are in agreement with previous reports demonstrating that growth with bile leads to induction of genes involved in cell wall homeostasis in other *Lactobacilli*^39,40^.

Several genes involved in cell wall biogenesis or remodeling and fatty acid (FA) utilization (Fig. 2A, Table 1) were also induced with a log_2_ fold change > 1. These genes are highly relevant for processes that may lead to EV biogenesis or EV release from the cell, particularly the enrichment of phospholipids in the cytoplasmic membrane and the localized degradation of peptidoglycan. The expression of several genes encoding putative peptidoglycan hydrolases (PGHs) was also induced during growth of *L. johnsonii* N6.2 with bile. These include a peptidase (T285_RS01325) herein referred to as *pepD*, a muramidase herein referred to as *murV* (T285_RS00820) and *sdp* (T285_RS00825) that encodes for Sh3b domain-containing lysin. Notably, a proteomics characterization of *L. johnsonii* N6.2 derived EVs previously identified the PepD and Sdp proteins to be enriched in the EVs relative to the cell membrane^12^. Western blot analysis using an antibody specific for the SH3b2 domain of Sdp (Sdp_SH3b2) revealed that Sdp is more abundant in EVs from *L. johnsonii* N6.2 grown in bile compared to the control EVs (Supplementary Fig. 5). These data combined emphasize the potential role of transcriptionally controlled PGH activity facilitating EV release.

**Table 1:** Differentially expressed genes relevant to cellular processes contributing to EV biogenesis.

	Locus tag	log2 Fold Change	padj	Gene name	Annotation
Putative peptidoglycan hydrolase genes	T285_RS00820	1.74	2.67E−27	*murV*	Muramidase
	T285_RS00825	1.38	1.00E−04	*sdp*	Sh3b domain containing lysin
	T285_RS01325	1.33	1.74E−09	*pepD*	Peptidase
Putative fatty acid metabolism genes	T285_RS04705	1.39	2.05E−08	*fakT*	MarR family transcriptional regulator
	T285_RS04710	2.66	1.67E−72	*fakB4*	DegV domain containing protein

The expression of multiple genes in the FA kinase pathway were induced, including two genes encoding DegV domain-containing proteins. These proteins have been shown in other gram-positive bacteria to bind exogenous FAs after they enter the cell for phosphorylation and downstream incorporation into membrane phospholipids^41,42^. The *Streptococcus pneumoniae* genome encodes three of these DegV domain-containing proteins named FakB1, FakB2, and FakB3. These proteins have been experimentally shown to bind FAs based on their degree of saturation; FakB1 preferentially binds saturated FAs (SFA), FakB2 preferentially binds monounsaturated FAs (MUFA), and FakB3 preferentially binds polyunsaturated FAs (PUFA)^42^. The *L. johnsonii* N6.2 genome encodes four FakB homologs. In presence of bile, the expression of T285_ RS04660 and T285_ RS04710 was induced. Protein sequence alignments of these two FakB proteins revealed that both share the highest identity to FakB1 from *S. pneumoniae* and will herein be referred to as FakB1 and FakB4, respectively. The other two genes encoding DegV domain containing proteins were not differentially expressed during growth with bile, and these were named FakB2 (T285_RS05090) and FakB3 (T285_RS00120) based on sequence identity to the *S. pneumoniae* FakB proteins.

Directly upstream of *fakB4* is a gene encoding a MarR-family transcriptional regulator (T285_RS04705), herein referred to as *fakT*, that was also induced (log_2_ FoldChange C = 1.39) during growth of *L. johnsonii* N6.2 with bile. The MarR family of transcription factors, which is found ubiquitously across bacteria, includes FabT, a transcription factor identified as a repressor of de novo fatty acid biosynthesis in gram-positive bacteria^43^. The *L. johnsonii* N6.2 genome, however, does not encode all the genes required for de novo FA synthesis and requires exogenous fatty acid supplementation for growth^44^. In the absence of a de novo synthesis pathway, *L. johnsonii* N6.2 is reliant on external sources of FAs to maintain membrane homeostasis and respond to external stressors^44^. Thus, we hypothesize that this transcription factor may be a regulator of exogenous fatty acid utilization, and the fatty acids in bile may be incorporated into the membrane favoring membrane budding and EV release.

**Figure 4:**
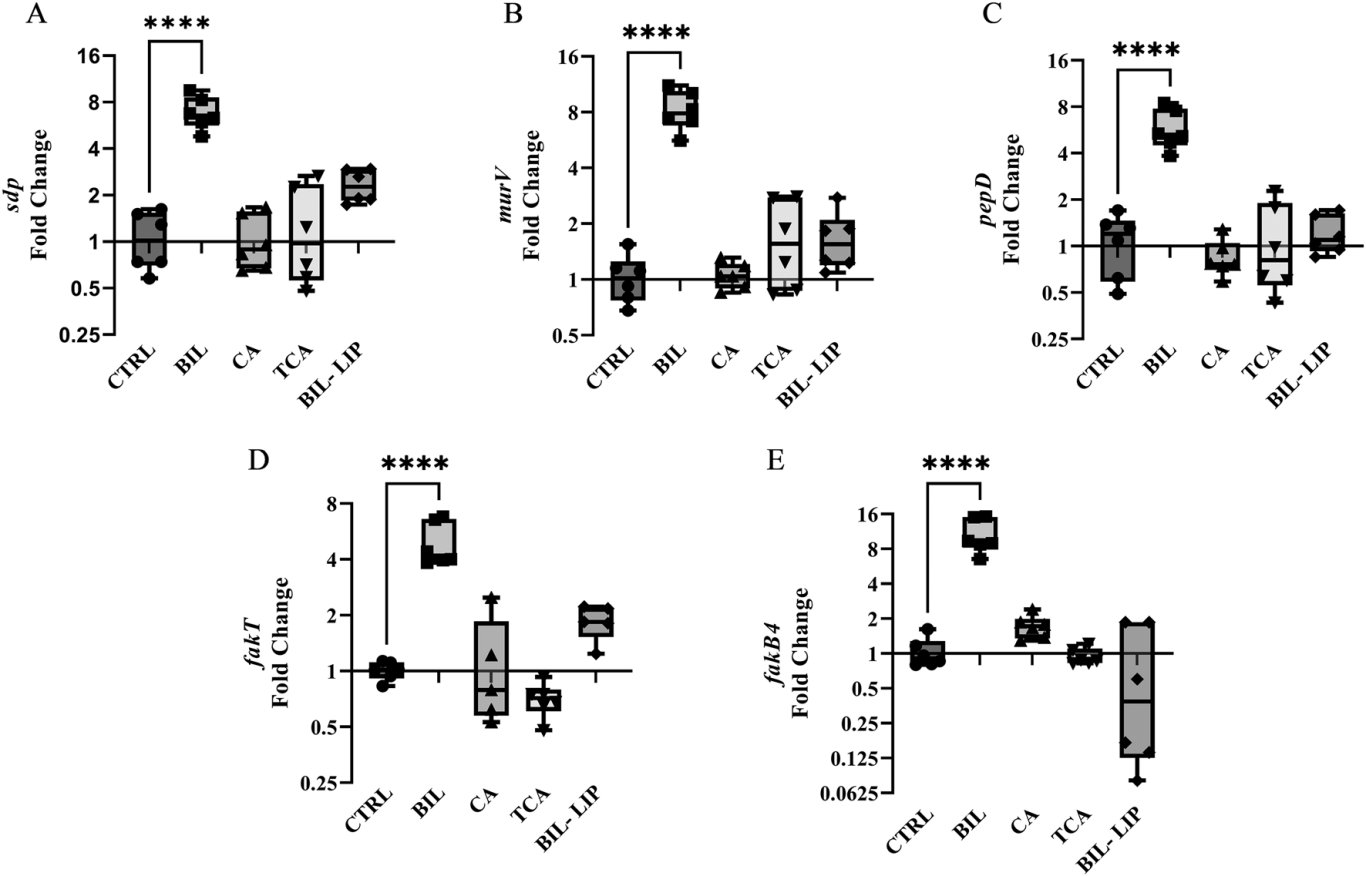
Bile induces the expression of genes involved in peptidoglycan remodelling and exogenous fatty acid utilization. Relative gene expression levels in *L. johnsonii* N6.2 grown with 0.2% bovine bile (BIL), 2.5 mM cholic acid (CA), 12.5 mM taurocholic acid (TCA), and 0.02% bile lipids (BIL-LIP). (**A**) *sdp*, (**B**) *murV*, (**C**) *pepD*, (**D**) *fakT*, and (**E**) *fakB4*. RNA was extracted from biological triplicates and qRT-PCR measurements for each triplicate were performed in technical duplicates. Significance was determined using a one way ANOVA with post-hoc Tukey HSD. *p < 0.05, **p < 0.01, ***p < 0.001, ****p < 0.0001.

To validate these findings, we compared the expression levels of *fakT*, *fakB4*, *pepD*, *murV*, and *sdp* in *L. johnsonii* N6.2 cells grown in bile to those grown with individual bile salts. It was found that while the addition of bile induced the expression of these genes, as expected, growth in presence of CA or TCA did not result in significant changes in expression pattern of the genes tested (Fig. 4A–E). These results are in agreement with the reduced formation of EVs described when cells were grown in presence of CA or TCA (Fig. 1A). Interestingly, just as bile lipids promoted a distinct yet insignificant increase in the number of EVs/CFU, a similar trend in induction in gene expression levels for *fakT* and *sdp* was observed. These results demonstrate a positive correlation between the expression levels of our genes of interest and the number of EVs produced by *L. johnsonii* N6.2. These results, along with the observed upregulation of these genes under a hypervesiculation-promoting growth condition, provides correlational evidence that these enzymes contribute to EV production. However, due to our inability to genetically modify *L. johnsonii* N6.2, we are unable to establish a direct causal relationship between the individual enzymes and the production of EVs.

### Bile-induced hypervesiculation stimulates similar responses in human βlox5 and THP-1 cell lines

**Figure 5:**
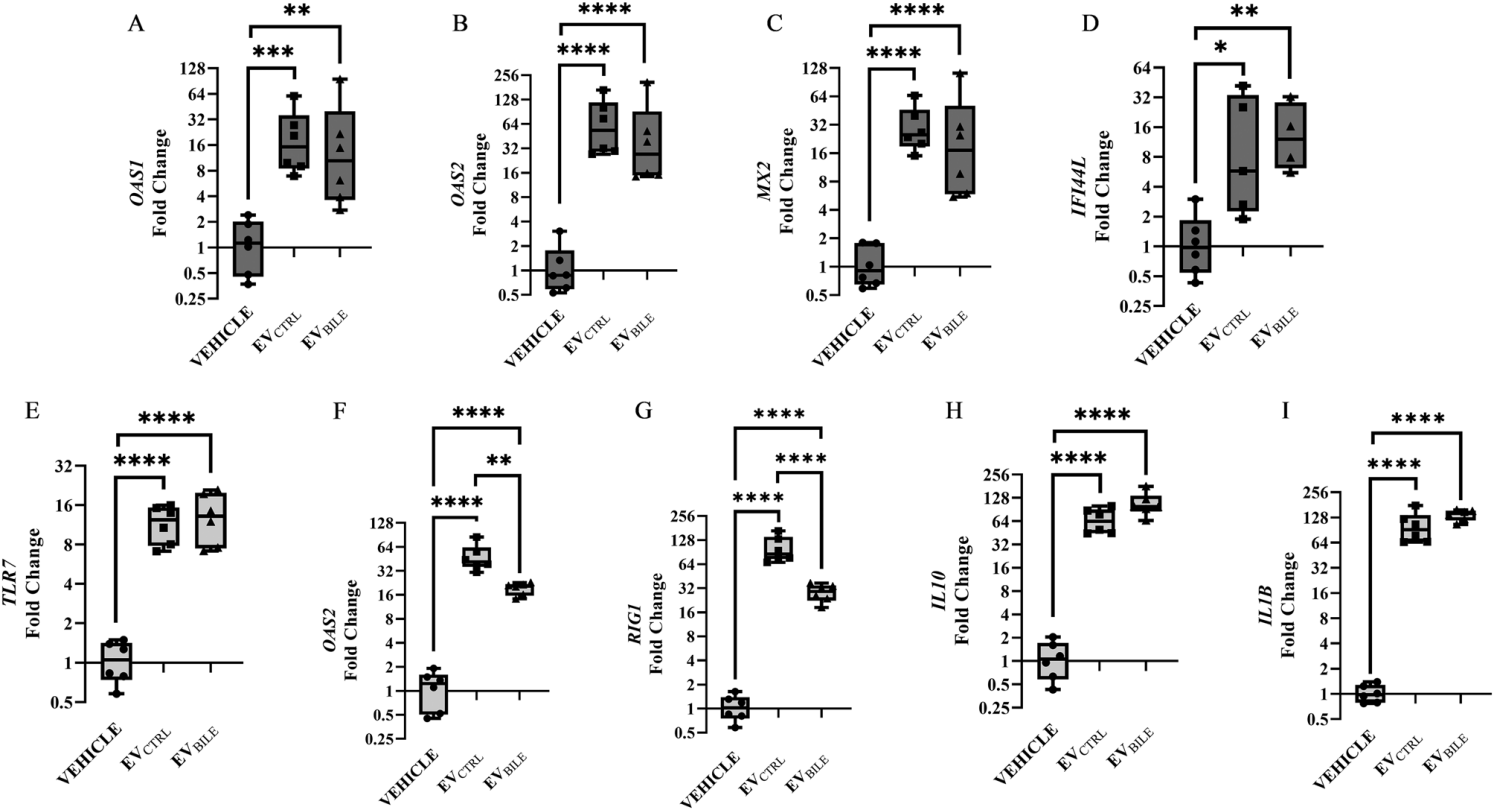
EV_CTRL_ and EV_BILE_ elicit a similar response in host cell lines. Relative gene expression levels of (**A**) *OAS1*, (**B**) *OAS2*, (**C**) *MX2*, and (**D**) *IFI44L* in βlox5 cells and (**E**) TLR7, (**F**) *OAS2*, (**G**) *RIG1*, (**H**) *IL10*, and (**I**) *IL1B* in THP-1 cells. Cells were treated for 6 h with 1 × 10^10^ EVs isolated from cultures grown in presence or in absence of 0.2% bile. Cells were treated in triplicate and qRT-PCR measurements for each triplicate were performed in duplicate. Significance was determined using a one way ANOVA with post-hoc Tukey HSD. *p < 0.05, **p < 0.01, ***p < 0.001, ****p < 0.0001.

We reported that in human pancreatic βlox5 cells, the addition of EVs at 10^10^ induce the expression of multiple genes involved in nucleic acid sensing and degradation, including the OAS genes, IFI44L, and MX2^5,45^. Next we evaluated whether EVs isolated from *L. johnsonii* N6.2 grown in the presence of bile (EV_BILE_) would elicit a similar response in host cell lines to EVs obtained from standard conditions (EV_CTRL_). To this end, βlox5 cells were treated for 6 h with EV_BILE_ and EV_CTRL_, and the expression of *OAS1*, *OAS2*, *MX2*, and *IFI44L* was followed with qRT-PCR. It was found that the treatment with EV_BILE_ resulted in a similar stimulation pattern as EV_CTRL_ for all the genes tested (Fig. 5A–D).

These results were further validated using THP-1 human macrophages. Similar to βlox5 cells, treatment with EVs at 10^10^ elicited a similar RNA sensing response in THP-1 cells^5^. Here, *TLR7*, *OAS2*, and *RIG1* were selected as markers for nucleic acid sensing and degradation. It was found that EV_BILE_ maintained the ability to significantly induce expression of RNA sensing and degradation genes relative to the PBS vehicle control, albeit to a lesser extent than EV_CTRL_ (Fig. 5E–G). Lastly, we evaluated the ability of both EV preparations to induce expression of the *IL10* and *IL1B* cytokines as markers of a M2b tolerogenic profile in macrophages. Cells treated with both EV_CTRL_ and EV_BILE_ showed a significant increase in gene expression, suggesting that both preparations are equally capable of promoting an anti-inflammatory environment in host cell lines (Fig. 5H,I).

## Discussion

EVs derived from a variety of probiotic strains are being increasingly studied for both their intrinsic therapeutic properties and their potential as engineered therapies that can be utilized to treat or prevent disease. Consequently, understanding mechanisms that influence their biogenesis is paramount to developing technologies that may maximize the beneficial interactions between a probiotic bacterium and its host. In this report, we identify bile as a physiologically relevant environmental factor that significantly increases the production of EVs by the probiotic bacterium *L. johnsonii* N6.2. We reported that *L. johnsonii* N6.2 EVs promote beneficial phenotypes in human pancreatic βlox5 and macrophage THP-1 cells, and that they can generate a host immune response at distal locations in vivo^5,12^. Thus, these EVs exhibit potential as therapies to preserve beta-cell function and mitigate T1D onset. EVs from other probiotics have recently been evaluated as therapeutics to treat or prevent disease^6,7,46^, but the practicality of these treatments is limited due in part to low yield and inefficient EV production and isolation methods. As *L. johnsonii* N6.2 derived EVs continue to be evaluated for their therapeutic capabilities, this report introduces a growth condition to scale up their production without compromising their immunomodulatory properties.

The results presented showed a direct correlation between the EV quantity produced by *L. johnsonii* N6.2 grown in bile the expression of specific genes. It was previously indicated that a potential explanation for the increase in EV biogenesis during growth with bile was that bile salts could induce charge repulsions between phospholipid molecules increasing membrane blebbing and EV release^47^. Here, we demonstrate that individual bile salts alone are insufficient in promoting vesiculogenesis. The lipid component of bile appears to be a contributing factor towards the increase in EV quantity, however lipids alone were not sufficient to recapitulate the hypervesiculation phenotype. These results suggest that the observed effect is a consequence of the additive impact of multiple bile components. Furthermore, these findings emphasize that the bile mediated increase in EV biogenesis cannot be attributed to disruptions in the cell envelope induced by bile acids. Together, it is proposed that EV release is an active response of the cell to bile or some of it’s components, suggesting a significant contribution from proteins whose expression is controlled at the transcriptional level.

Sdp, PepD, MurV, FakB4, and FakT were identified as potential genetic drivers of EV biogenesis. The increased expression of these genes in presence of bile was positively correlated with increased EV production in *L. johnsonii* N6.2. The enrichment of PGH proteins during proteomic analyses of EVs have been reported in several lactobacilli^6,7,10^. In general, two mechanisms have been proposed for PGH mediated hypervesiculation phenotype. In several gram-positive bacteria, such as *Bacillus subtilis*, *Lactococcus lactis* and *Lacticaseibacillus casei*, the induction of a prophage has been correlated with increased production of EVs^20–22^. The activity of prophage-encoded PGHs resulted in localized degradation the host peptidoglycan promoting the release of phage particles while the production of extracellular vesicles was deemed as an indirect effect. In *L. johnsonii* N6.2, the strong chromosomal localization of genes upregulated by the presence of bile initially led us to evaluate the induction of a temperate prophage, however our results indicated that the prophages encoded in the genome is incomplete and non functional.

The role of PGHs in EV biogenesis in other *Lactobacilli* has also been proposed independently of prophage induction, primarily based on the observation that proteins involved in peptidoglycan remodeling and degradation are commonly enriched in EVs relative to the cell membrane or whole cell fractions^6,7,10^. In agreement with these findings, proteomics analysis showed the *L. johnsonii* N6.2 EVs were enriched with two putative PGHs, Sdp and PepD, relative to the cell membrane^12^. Interestingly, the genes encoding these proteins were transcriptionally induced when *L. johnsonii* N6.2 was cultivated with bile, providing a potential mechanism for the observed increase in EV biogenesis. In a recent study, overexpression of the Msp1 PGH from *Lacticaseibacillus rhamnosus* was identified as a driver of EV production in *Escherichia coli* and *Bacillus subtilis*^48^. However, this system led to less than a tenfold increase in EV production in all strains tested, whereas cultivation with bile led to almost a 100-fold increase in EVs produced by *L. johnsonii* N6.2. We have presented a method to increase EV biogenesis independently of genetic modification, providing a strategy that may be applied to other lactobacilli wild type strains that are not amenable to genetic manipulation.

Another pathway proposed in EV biogenesis is the fatty acid kinase pathway, which is involved in the utilization of exogenous fatty acids for synthesis of membrane phospholipids. Localized Phospholipid enrichment has been proposed as a driver of vesiculogensis, as the accumulation of negative charge creates an electrostatic repulsion that favors membrane curvature and EV budding^9^. This assertion is supported by the lipid composition of *L. johnsonii* N6.2 EVs, which was shown to be enriched with cardiolipins, glyceryphosphoethanolamines, and glycerophosphoglycerols compared to the cell membrane^12^. In this report, we show that bile induces expression of two genes encoding FakB proteins that show high sequence similarity to FakB1 from *S. pneumonia*, which preferentially binds SFA for phosphorylation by FakA^42^. Many reports have described the effects of bile on the physiology of probiotic bacteria to assess their ability to colonize or, at minimum, survive passage through the small intestine to elicit a beneficial response within the host. For example, one previous study demonstrated that growth of *Bifidobacterium animalis* IPLA4549 with bile resulted in an increased ratio of saturated to unsaturated fatty acids in their membrane. These changes were associated with reduced membrane fluidity and lower permeability^49^. The increased expression of genes encoding FakB1 homologs suggest that a similar trend would be present in the membrane of *L. johnsonii* N6.2 when grown with bile, and that an increase in saturated:unsaturated FAs would favor EV budding and release. In agreement with these observations, exogenous addition of palmitic acid (16:0) was shown to increase the quantity of EVs produced by *Bacteroidetes fragilis* ATCC 23745^50^. The membrane physiology of *L. johnsonii* N6.2 is especially sensitive to exogenous fatty acids introduced during growth with bile, as this bacterium is not capable of de novo fatty acid synthesis and relies on exogenous fatty acids for synthesis of membrane phospholipids^44^. While the membrane composition of *L. johnsonii* N6.2 and it’s EVs was not elucidated in the current study, the increase in EV production induced by bile in combination with gene expression data implies that the lipid constituents of bile influence the physiology of the membrane in a manner conducive to EV budding.

This work demonstrates that current methods for quantifying EVs from bacteria are not always representative of the actual physiological changes influencing EV production. For example, EVs are often quantified as EVs/mL of solvent and normalized to optical density, but not live cells in the culture. However, changes in cell morphology can affect optical density readings in ways that are not always reflective of cell density. Therefore, it is important to normalize EV counts to viable bacteria within the culture, and to cell length or circumference. In the case of mitomycin C, the elongation of bacterial cells led to an increase in the number of EVs/CFU. This artifact was created by the increased cell length and not as a consequence of transcriptionally controlled increase in protein expression leading to increased vesiculation, as was observed in the presence of bile. Other normalization methods that also account for the number of live cells in culture could also be explored. For example, strains amenable to genetic manipulation could be engineered to produce GFP, and EV count could be normalized to fluorescence.

In previous reports, it was observed that cultivating the *Lacticaseibacillus casei* strain at pH 8 or growing *Lactiplantibacillus plantarum* under static conditions resulted in a significant increase in the production of EVs compared to the reference^23^. However, these EV preparations failed to induce an anti-inflammatory phenotype in THP-1 macrophages, consequently limiting their potential therapeutic applications. In contrast to these results, we showed that EVs derived from *L. johnsonii* N6.2 grown in a hypervesiculation-inducing condition elicit a similar tolerogenic host response in βlox5 and THP-1 human cell lines compared to EVs grown under standard conditions. These data suggest that, despite global changes in gene expression that occur when this strain is grown with bile, the bioactive components responsible for interacting with host cells are still produced at adequate levels and packaged into or on the surface of the EVs. The conserved immune response also confirms that EV_BILE_ are internalized by the host cell. It is currently unclear whether an active sorting mechanism is necessary for the inclusion of immunomodulatory components within EVs, or if these components are simply present due to their proximity to sites of EV release. Recently, *L. johnsonii* N6.2 EVs were shown to be enriched with RNA transcripts encoding membrane bound and extracellular proteins compared to total cell RNA^45^. These data suggest that transcription and translation coupling drives RNA packaging into EVs. Additional experiments are needed to ascertain if this mechanism also applies to proteins and other immunomodulatory components. Taken together, our results are significant in order to evaluate the potential therapeutic applicability of inducing hypervesiculation in *L. johnsonii* N6.2.

In summary, we have identified bile as a potent inducer of EV production by *L. johnsonii* N6.2. Additionally, we identified several genes whose expression is induced by bile and may serve potential markers for the stimulation of EV release during technological applications. These findings are significant because they indicate that vesiculogenesis may be partly mediated by proteins whose expression is controlled at the transcriptional level, suggesting the possibility that this process may be controlled using small molecules. However, further experiments using a strain amenable to genetic manipulation will be required to establish a direct causational link between individual genes and the biogenesis of EVs. Furthermore, production of EVs was increased without compromising their ability to elicit an RNA sensing response in human pancreatic beta cells or a tolerogenic anti-inflammatory response in macrophages. As probiotic-derived EVs continue to be evaluated for their therapeutic properties, additional research will be needed to identify strategies to control their production and maximize the beneficial properties of probiotics and their effectors in vivo.

## Methods

### *L. johnsonii* N6.2 growth conditions

*L. johnsonii* N6.2 was routinely grown in de Man, Rogosa, Sharpe (MRS) broth at 37 °C. Vesicle-depleted MRS (vdMRS) was utilized for growth curves and EV isolation^12^. Breifly, 5 × MRS media was ultracentrifuged 175,000×*g* for 2 h, at 4 °C, diluted to 1 ×, then filter sterilized through a 0.2 µm filter. vdMRS was supplemented with rosmarinic acid (100 µM RA), vesicle-depleted oxgall (0.05–0.2% bile), cholic acid (2.5–5 mM CA), taurocholic acid (5–25 mM TCA), glycocholic acid (5–15 mM GCA), deoxycholic acid (2.5–7.5 mM DCA), taurodeoxycholic acid (2.5–15 mM TDCA), glycodeoxycholic acid (0.5–2.5 mM GDCA), and mitomycin C (58 nM–936 nM MMC). All media was adjusted to pH 6.5 prior to inoculation. For each condition, cultures were grown under static conditions at 37 °C, except for the aerobic condition in which *L. johnsonii* N6.2 was grown shaking at 75 rpms at 37 °C. A single concentration of each component with a negative but sub-inhibitory effect on growth was selected for its effect on EV production: 75 rpms (AER), 0.2% bovine bile (BIL), 2.5 mM CA, 12.5 mM TCA, and 7.5 mM DCA, and 116.7 nM MMC. For RA, TCA, and bile lipids that did not have a negative impact on growth at the highest physiologically relevant concentrations, the highest relevant concentration was used. EVs were isolated in biological triplicate from 250 mL bacterial cultures as previously described^12^.

### Isolation and quantification of *L. johnsonii* N6.2 EVs

*L. johnsonii* N6.2 cultures were grown in triplicates for each condition until late exponential phase. Colony forming units (CFU/mL) were determined by performing serial dilutions in sterile 0.9% peptone water. These serial dilutions were plated on MRS pH 6.5 agar plates and incubated under microaerophilic conditions at 37 °C for 48 h. The remaining culture was centrifuged at 20,000 × g for 20 min, at 4 °C. The supernatant was passed through a 0.2 µm filter to remove any remaining bacterial cells. The filtered supernatant was ultracentrifuged 175,000×*g* for 2 h, at 4 °C, and the resulting pellet was washed once with PBS following the same ultracentrifugation parameters. The washed EV pellet was resuspended in 500 µL PBS and immediately subject to nanoparticle tracking analysis (NTA) quantified using Nanosight NS300 (Malvern instruments Ltd, Malvern, UK) to measure particle/mL and EV diameter as previously described^12^. Aliquots were preserved at − 80 °C. EV concentration was expressed as EV/mL of bacterial culture. To determine EVs/CFU, EVs per mL of bacterial culture was divided by CFU/mL of culture to yield a value for EVs/CFU.

### Total lipid extractions

A 10% solution of bovine bile (Sigma Aldrich, B3883) dissolved in water was ultracentrifuged at 175,000×*g* for 2 h at 4 °C to remove any bile-derived EVs. This solution was then lyophilized and stored at 4 °C in the dark for later lipid extractions. Total lipids from bile were extracted using a modified Bligh and Dyer method^51^. In short, two grams of EV depleted freeze-dried bile were added into a clean glass separatory funnel. A total of 228 mL of ACS or HPLC grade solvents were added in sequence to achieve a final cloroform:methanol:water ratio of 1:2:0.8 (v/v/v). The mixture was allowed to stand for 18 h with occasional shaking and subsequent phase separation was achieved by adding chloroform and water to reach a chloroform:methanol:water ratio of 1:1:0.9 (v/v/v). The lower chloroform phase was collected. Most of the solvent was evaporated in a rotavapor (Buchi, New Castle, DE) at 474 mbar and a water bath of 40 °C. The remaining solvent was evaporated under a nitrogen stream and dried lipid extractions were sealed in glass tubes and stored at − 80 °C. Lipids were solubilized by adding vdMRS and heating to 37 °C prior to filter sterilization (0.22 μm).

### Brightfield and scanning electron microscopy

All electron microscopy images were obtained at the Microscopy Core Interdisciplinary Center for Biotechnology Research (University of Florida, RRID: SCR_019146). Briefly, *L. johnsonii* N6.2 cell pellets were fixed with Trump’s fixative solution (VWR, USA), placed onto 0.2 μm polycarbonate membrane and processed using a Pelco BioWave Pro laboratory microwave (Ted Pella, Redding, CA, USA). The samples were washed three times with PBS pH 7.2 and post-fixed with 1% buffered osmium tetroxide. After the post-fixation step, the samples were washed with distilled water and dehydrated with a graded ethanol series (25%, 50%, 75%, 95%, 100%). The surface structures of the samples was preserved by drying them in a critical point dryer (Autosamdri-815, Tousimis Research Corp, Rockville MD, USA). The samples were mounted on carbon adhesive tabs on aluminum specimen mounts and Au/Pd sputter coated (DeskV Denton Vacuum, Moorestown, NJ, USA). Digital micrographs were acquired by field-emission scanning electron microscope (SU-5000, Hitachi High Technologies America, Inc. Schaumburg, IL, USA).

Brightfield microscope images were collected using an Olympus BX41 polarizing light microscope at 100 × magnification at late exponential phase. ImageJ^52^ was used to measure cell lengths of at least 30 cells from each group, and this information was used to calculate the average cell length for each condition. The EVs/CFU values previously determined were divided by the average cell length from each respective condition to yield a value for EVs per µm per CFU.

### DNA and RNA extractions

DNA was extracted from *L. johnsonii* N6.2 cells grown to late exponential phase using the DNeasy Blood & Tissue kit (QIAGEN, Germantown, MD) following the manufacturer’s protocol for gram-positive bacteria. For RNA extractions, *L. johnsonii* N6.2 was cultured under each of the previously described conditions until mid-exponential growth phase. Cells were harvested by centrifugation at 20,000×*g* for 20 min, at 4 °C, and pellets were immediately frozen at -80 °C until RNA extractions were performed. Briefly, 100 mg *L. johnsonii* N6.2 pellets were resuspended in Trizol and the suspension was added to 100 mg ice-cold zirconia beads. Cell lysis was performed by vortexing each tube for 5 30-s increments, resting the tubes on ice in between each round of vortexing. To remove the beads, the mixtures were centrifuged at 12,000×*g* for 10 min at 4 °C, and the supernatant was transferred to a fresh tube and incubated at room-temperature for 5 min. Next, 0.2 × volume of chloroform was added followed by manual shaking for 15 s, and then a 10-min incubation at room temperature. The samples were centrifuged at 12,000×*g* for 15 min at 4 °C, and the top aqueous phase was transferred to a fresh tube. Then, 1 × volume of 100% ethanol was added, and the mixture was loaded into spin columns from the RiboPure-Bacteria kit (Invitrogen, Waltham, MA). Subsequent wash steps were performed following the manufacturer’s instructions. RNA was also routinely isolated from human cell lines using the Qiagen Rneasy Miniprep following manufacture’s specifications (QIAGEN, Germantown, MD). DNA contamination was removed using the Invitrogen TURBO DNase kit following the manufacturers recommendations. RNA integrity was examined on 1% agarose gels, and RNA quantification was performed using Thermo Scientific Nanodrop One Microvolume UV–Vis spectrophotometer (Thermo Fisher Scientific, Grand Island, NY).

### RNA-seq analysis

Library construction and sequencing was performed by Novogene, (Novogene Co., Davis, CA, USA) as described earlier^5^. After cluster generation, the library preparations were sequenced on an Illumina platform and paired-end reads were generated. Raw data (raw reads) of FASTQ format were first processed through fastp. The reference genome [NC_022909.1^53^] and gene model annotation files were downloaded from NCBI. Bowtie2 was used to build the index of the reference genome and to align the clean reads^54^. FeatureCounts was used to count the read numbers mapped to each gene^55^. Differential expression analysis was performed using the DESeq2 R package, and the resulting p-values were adjusted using Benjamini and Hochberg’s approach for controlling the false discovery rate^56,57^. Genes with an adjusted p-value < 0.05 found by DESeq2 were assigned as differentially expressed. Gene Ontology (GO) enrichment analysis of differentially expressed genes was implemented by the clusterProfiler R package, in which gene length bias was corrected^58^. GO terms with a corrected p-value less than 0.05 were considered significantly enriched by differential expressed genes. The clusterProfiler R package was used to test the statistical enrichment of differential expression genes in KEGG pathways^59–62^.

### PCR and qRT-PCR analysis

Total DNA and RNA extraction were performed from *L. johnsonii* whole cells as described above. PCR reactions were conducted using the Q5 2 × master mix (New England Biolabs, Ipswich, MA) following the manufacturer’s recommendations. cDNA was synthesized using iScript cDNA Synthesis Kit (Bio-Rad, Hercules, CA, United States) following manufacturer instructions. The qRT-PCR assays were performed using PowerUp SYBR Green Master Mix (Applied Biosystems, Foster City, CA, United States) in a QuantStudio 6 machine (Applied Biosystems, Foster City, CA, United States). Residual DNA contamination was tested by PCR amplification of the 16S rRNA gene from RNA samples. The changes in gene expression (Ct values) between treatments controls were compared using the 2^–ΔΔCt^ method. The expression of the 16S rRNA gene was used as an internal control for *L. johnsonii* N6.2, and GAPDH was used for human. The primers used are described in Table 2.

**Table 2:** Primers used for all PCR, qPCR, and qRT-PCR experiments.

Primer name	Organism	Primer sequence (5ʹ–3ʹ)	Source
1	*L. johnsonii* N6.2	TCTCTGCACCAGCAATCAGG	*
2	*L. johnsonii* N6.2	CCTCCGGTGAAGTTTCTCGT	*
3	*L. johnsonii* N6.2	GCTCGAGTAATTTGGTTATTGGTCA	*
4	*L. johnsonii* N6.2	CGCCCAACTACTGGGAACAA	*
5	*L. johnsonii* N6.2	TCTACCGTTGACTGGTTGGC	*
6	*L. johnsonii* N6.2	CAATGCGATGGAGCGTCAAG	*
7	*L. johnsonii* N6.2	TCAAAGCTGCAGAAGAAAAGTT	*
8	*L. johnsonii* N6.2	GCTCGAGTAATTTGGTTATTGGTCA	*
9/fakT_F	*L. johnsonii* N6.2	AGCAGAAGAAAACTGCGTCT	*
10/fakT_R	*L. johnsonii* N6.2	TGTGCTTCAATTTGAGCTAGAAC	*
murV_F	*L. johnsonii* N6.2	ACAACCAAGCTAGCCAACCA	*
murV_R	*L. johnsonii* N6.2	GTCGTAGTTTGGGCTGGAGT	*
pepD_F	*L. johnsonii* N6.2	AGGTGTAGAACGCGTAGGATATT	*
pepD_R	*L. johnsonii* N6.2	CGCATCATCTGGAATACGGC	*
sdp_F	*L. johnsonii* N6.2	GCCTACTACTGATACTAACAACACC	*
sdp_R	*L. johnsonii* N6.2	TGGATTTGCAGGTGCTTGAC	*
fakB4_F	*L. johnsonii* N6.2	CGATGGAGCACCTTTTCCTA	*
fakB4_R	*L. johnsonii* N6.2	AACGGTCATTGCTGGATCAT	*
16S_F	*L. johnsonii* N6.2	GCCTAGATGATTTTAGTGCTTGCA	*
16S_R	*L. johnsonii* N6.2	GCAGGTTACCCACGTGTTACTCA	*
OAS1_F	Human	AACCTCACACTGGTTGGCAG	*
OAS1_R	Human	CTGCCTCCCTAAGCAACCTG	*
OAS2_F	Human	ACCCGAACAGTTCCCCCTGGT	^45^
OAS2_R	Human	ACAAGGGTACCATCGGAGTTGCC	^45^
MX2_F	Human	CATGCATCAGGGGTCCACAC	^45^
MX2_R	Human	GGACTGGAGAGCCATCCCTT	^45^
IFI44L_F	Human	CAGCTTCCACGTGTGAGTGAG	^45^
IFI44L_R	Human	ACGGCTGCATCTTTCAACCA	^45^
GAPDH_F	Human	GAAGGTGAAGGTCGGAGTC	^5^
GAPDH_R	Human	GAAGATGGTGATGGGATT	^5^
TLR7_F	Human	CAATTGCTTCCGTGTCATCCAG	^5^
TLR7_R	Human	CCCTATGGAAACCCAGAAGCAG	^5^
RIG1_F	Human	TAAGGGGATGATGGCAGGTG	*
RIG1_R	Human	TGGGCCAGTTTTCCTTGTCT	*
IL10_F	Human	CCAAAATCGGATCTGGGGCT	^5^
IL10_R	Human	GGGGGTTGAGGTATCAGAGG	^5^
IL1B_F	Human	CAGAAGTACCTGAGCTCGCC	^5^
IL1B_R	Human	AGATTCGTAGCTGGATGCCG	^5^

*Original to this work.

### Western Blot analysis

Western blot using anti-Sdp_SH3b2 was performed as previously described^12^. EVs were loaded at 2.2 × 10^9^ for both EV preparations, and 10 ng his-tagged Sdp_SH3b2 was obtained from prior work as a positive control.

### Culture and treatment of human cell lines

The pancreatic cell line βlox5 was obtained from Dr. Fred Levine (Sanford Children’s Health Research Center, La Jolla, CA)^63^. The human monocyte THP-1 cell line was obtained from ATCC (Gaithersburg, MD, USA). βlox5 pancreatic cells were cultured as previously described^5^. For EV treatment assays, βlox5 cells were seeded at 3 × 10^5^ cells per well in a 6 well plate and incubated overnight at 37 °C in a 5% CO_2_ incubator. Subsequently, EVs resuspended in the cell culture media were added at 1 × 10^10^ EVs per well, and an equivalent volume of media was added to the vehicle control. Cells were incubated with the EVs for 6 h followed by immediate RNA extractions. THP-1 cells were cultured in RPMI 1640 medium supplemented with 10,000 units of penicillin, 10 mg/mL streptomycin, and 10% heat inactivated FBS at 37 °C in a 5% CO_2_ incubator. Naïve monocytes were seeded in 6 well plates at 5 × 10^5^ cells/well and activated by adding phorbol 12-myristate-13-acetate (PMA) at 100 nM, for 48 h, at 37 °C. The culture media was removed, cells were washed with 1 mL sterile PBS (pH 7.2). EVs resuspended in PBS were added to the media at 1 × 10^10^ EVs per well, or an equal volume of PBS was added as a vehicle control. The plates were incubated for 6 h followed by immediate RNA extractions.

### Statistical analyses

R studio (RStudio Team, Boston, MA) and GraphPad Prism 5.01 software (GraphPad Software, La Jolla, CA, United States) were used for data analysis and visualization. Statistical tests were performed using one-way analysis of variance (ANOVA) and Tukey’s HSD post hoc test unless otherwise described. For all experiments, p ≤ 0.05 was considered statistically significant, and α = 0.05 was used for Tukey’s HSD tests.

### Supplementary Information

Supplementary Information.

## Data Availability

The datasets presented in this study can be found in the NCBI Sequence Read Archive online repository. The BioProject number is PRJNA964723.
