# Supplementary material for: Bile promotes Lactobacillus johnsonii N6.2 extracellular vesicle production with conserved immunomodulatory properties
Source: Sci Rep. 2024 May 28;14:12272. doi: 10.1038/s41598-024-62843-0 (PMC11133329; doi:10.1038/s41598-024-62843-0)
Supplement: Supplementary file 1 — Supplementary Information. [file 41598_2024_62843_MOESM1_ESM.pdf]

Supplementary Materials

Bile promotes *Lactobacillus johnsonii* N6.2 extracellular vesicle production with conserved immunomodulatory properties

Reagan E. Beliakoff[1], Claudio F. Gonzalez[1] & Graciela L. Lorca[1*]

[1] Department of Microbiology and Cell Science, Genetics Institute, Institute of Food and Agricultural Sciences, University of Florida, Gainesville, Florida, USA.

***Corresponding author:**
Graciela L. Lorca
Tel: +1 (352) 273 8090
Fax: +1 (352) 273 8284
e-mail: glorca@ufl.edu

Supplementary Table 1: Genes induced by bile with a log_2FoldChange ≥ 1 and padj < 0.05

**Locus tag**	**log2 Fold Change**	**padj**	**Gene name**	**Annotation**
T285_RS04930	5.54	7.23E-69	-	hypothetical protein
T285_RS09035	5.03	3.63E-06	-	hypothetical protein
T285_RS04865	4.86	6.90E-22	-	hypothetical protein
T285_RS09270	3.71	1.38E-102	-	zinc-binding dehydrogenase
T285_RS04630	3.68	1.05E-120	*recU*	Holliday junction resolvase RecU
T285_RS04285	3.50	5.30E-13	*pyrR*	bifunctional pyr operon transcriptional regulator/uracil phosphoribosyltransferase PyrR
T285_RS04625	3.48	1.12E-91	-	penicillin-binding protein
T285_RS04565	3.37	5.81E-85	*mvaD*	diphosphomevalonate decarboxylase
T285_RS04560	3.25	3.22E-183	-	phosphomevalonate kinase
T285_RS04570	3.24	4.72E-129	*mvk*	mevalonate kinase
T285_RS04655	3.24	3.92E-45	-	acyltransferase family protein
T285_RS04270	3.20	1.58E-08	-	dihydroorotase
T285_RS04275	3.19	1.11E-08	*carA*	glutamine-hydrolyzing carbamoyl-phosphate synthase small subunit
T285_RS04280	3.08	9.14E-24	*carB*	carbamoyl-phosphate synthase large subunit
T285_RS04555	3.08	2.04E-116	-	type 2 isopentenyl-diphosphate Delta-isomerase
T285_RS04635	3.08	1.02E-19	-	DUF1273 domain-containing protein
T285_RS04575	3.04	5.12E-38	-	ATP-dependent helicase
T285_RS04740	3.03	4.29E-50	*recQ*	DNA helicase RecQ
T285_RS04580	3.02	1.64E-193	*addA*	helicase-exonuclease AddAB subunit AddA
T285_RS04240	2.97	1.88E-116	-	DUF2974 domain-containing protein
T285_RS04265	2.95	1.17E-08	-	aspartate carbamoyltransferase catalytic subunit
T285_RS04730	2.87	2.15E-20	-	DUF1275 domain-containing protein
T285_RS04455	2.73	1.58E-10	-	GNAT family N-acetyltransferase
T285_RS04330	2.72	2.02E-07	-	hypothetical protein
T285_RS04585	2.72	1.68E-138	-	DEAD/DEAH box helicase family protein
T285_RS04590	2.70	5.52E-100	-	hypothetical protein
T285_RS04660	2.67	1.07E-40	-	DegV family protein

T285_RS04260	2.66	5.95E-22	*pyrR*	bifunctional pyr operon transcriptional regulator/uracil phosphoribosyltransferase PyrR
T285_RS04710	2.66	1.67E-72	-	DegV family protein
T285_RS04645	2.61	7.76E-65	-	class I SAM-dependent RNA methyltransferase
T285_RS04335	2.58	2.30E-13	-	glycerate kinase
T285_RS04650	2.56	9.91E-55	-	metallophosphoesterase
T285_RS04510	2.50	8.09E-13	-	ASCH domain-containing protein
T285_RS09250	2.48	4.82E-06	-	hypothetical protein
T285_RS04370	2.47	6.04E-08	-	MFS transporter
T285_RS04505	2.47	5.79E-15	-	nucleoside phosphorylase
T285_RS04685	2.47	1.12E-36	-	carbamoyl phosphate synthase small subunit
T285_RS04245	2.46	9.83E-07	-	orotate phosphoribosyltransferase
T285_RS03875	2.46	2.31E-61	-	phosphatase PAP2 family protein
T285_RS04305	2.45	1.91E-18	-	DNA alkylation repair protein
T285_RS04100	2.42	4.49E-36	-	glycine--tRNA ligase subunit beta
T285_RS04525	2.41	2.39E-09	*rbsD*	D-ribose pyranase
T285_RS04230	2.40	3.67E-17	-	DUF218 domain-containing protein
T285_RS04490	2.40	2.50E-14	-	phosphotransferase
T285_RS04715	2.40	4.44E-29	-	hypothetical protein
T285_RS04690	2.38	9.42E-124	-	carbamoyl phosphate synthase large subunit
T285_RS04765	2.37	9.55E-08	*plsY*	glycerol-3-phosphate 1-O-acyltransferase PlsY
T285_RS01130	2.35	4.39E-45	-	glycoside hydrolase family 65 protein
T285_RS04720	2.34	5.92E-95	-	LysR family transcriptional regulator
T285_RS04680	2.31	8.54E-73	-	RluA family pseudouridine synthase
T285_RS04315	2.30	3.18E-51	-	ABC transporter permease
T285_RS04745	2.29	4.68E-55	-	manganese-dependent inorganic pyrophosphatase
T285_RS04540	2.28	2.32E-58	-	MFS transporter
T285_RS04755	2.27	9.05E-71	*parC*	DNA topoisomerase IV subunit A
T285_RS04295	2.26	4.59E-175	*ppc*	phosphoenolpyruvate carboxylase
T285_RS04290	2.25	1.01E-40	-	cysteine hydrolase
T285_RS04530	2.24	9.61E-22	-	ribose transporter RbsU
T285_RS09245	2.23	3.06E-11	-	MFS transporter
T285_RS04600	2.22	2.44E-12	-	DnaD domain protein
T285_RS04105	2.21	1.31E-86	-	DNA primase

T285_RS04670	2.21	1.47E-63	-	formate--tetrahydrofolate ligase
T285_RS04095	2.20	1.77E-87	*glyQ*	glycine--tRNA ligase subunit alpha
T285_RS04700	2.18	1.51E-127	-	fibronectin/fibrinogen-binding protein
T285_RS04235	2.17	6.43E-33	-	Cof-type HAD-IIB family hydrolase
T285_RS04675	2.16	2.64E-47	-	signal peptidase II
T285_RS04770	2.15	6.68E-11	-	sugar O-acetyltransferase
T285_RS04255	2.15	3.37E-22	-	dihydroorotate dehydrogenase
T285_RS04780	2.15	6.79E-23	-	ABC transporter ATP-binding protein
T285_RS04015	2.14	8.21E-25	*aspS*	aspartate--tRNA ligase
T285_RS04785	2.11	1.71E-09	-	ABC transporter permease
T285_RS04220	2.11	4.36E-59	-	NOL1/NOP2/sun family putative RNA methylase
T285_RS04440	2.09	9.17E-19	-	hypothetical protein
T285_RS04515	2.09	1.12E-10	-	bifunctional 5%2C10-methylene-tetrahydrofolate dehydrogenase/5%2C10-methylene-tetrahydrofolate cyclohydrolase
T285_RS04110	2.08	1.57E-109	*rpoD*	RNA polymerase sigma factor RpoD
T285_RS04215	2.08	8.82E-46	-	hypothetical protein
T285_RS04725	2.07	9.76E-25	*xerS*	tyrosine recombinase XerS
T285_RS04310	2.07	1.02E-08	-	ABC transporter ATP-binding protein
T285_RS00460	2.06	1.35E-28	-	ABC transporter substrate-binding protein/permease
T285_RS04415	2.05	1.88E-04	-	HIT family protein
T285_RS04485	2.03	3.86E-10	-	kinase
T285_RS04640	2.03	1.43E-10	-	DivIVA domain-containing protein
T285_RS04065	2.02	1.42E-14	-	30S ribosomal protein S21
T285_RS04460	2.00	1.45E-06	-	NUDIX hydrolase
T285_RS04595	1.99	1.16E-43	*asnS*	asparagine--tRNA ligase
T285_RS04210	1.98	9.01E-37	-	hypothetical protein
T285_RS04735	1.96	1.57E-13	-	peptidylprolyl isomerase
T285_RS03975	1.92	3.65E-08	-	hypothetical protein
T285_RS04090	1.92	4.94E-16	*recO*	DNA repair protein RecO
T285_RS04535	1.91	3.29E-10	-	AAC(3) family N-acetyltransferase
T285_RS04010	1.91	4.48E-07	-	histidine--tRNA ligase
T285_RS04480	1.90	1.09E-06	-	antitoxin RelB
T285_RS04410	1.90	8.75E-04	-	GNAT family N-acetyltransferase
T285_RS04760	1.89	2.59E-54	*parE*	DNA topoisomerase IV subunit B
T285_RS04450	1.89	7.18E-06	-	GNAT family N-acetyltransferase
T285_RS04430	1.88	1.02E-10	-	tyrosine-protein phosphatase

T285_RS04500	1.86	5.27E-11	-	nucleoside phosphorylase
T285_RS04320	1.85	1.88E-66	-	M13 family metallopeptidase
T285_RS04145	1.85	1.03E-33	*pepT*	peptidase T
T285_RS04425	1.83	9.04E-05	-	hypothetical protein
T285_RS09005	1.82	3.04E-90	-	ABC transporter ATP-binding protein
T285_RS03805	1.80	1.68E-14	*dnaK*	molecular chaperone DnaK
T285_RS03890	1.78	8.24E-09	-	ATP-binding cassette domain-containing protein
T285_RS04135	1.78	1.15E-22	-	SAM-dependent methyltransferase
T285_RS03925	1.78	1.12E-09	-	ABC transporter permease
T285_RS04790	1.78	1.44E-08	-	hypothetical protein
T285_RS03940	1.78	1.39E-12	-	L-lactate dehydrogenase
T285_RS04170	1.76	6.78E-18	-	hypothetical protein
T285_RS03020	1.76	5.25E-07	-	GntR family transcriptional regulator
T285_RS00820	1.74	2.67E-27	-	hypothetical protein
T285_RS09025	1.74	1.67E-05	-	substrate-binding domain-containing protein
T285_RS09050	1.72	8.44E-07	-	DUF1542 domain-containing protein
T285_RS04605	1.71	1.07E-10	*nth*	endonuclease III
T285_RS07165	1.70	1.49E-02	-	rhodanese-like domain-containing protein
T285_RS03410	1.70	1.33E-05	-	Asp23/Gls24 family envelope stress response protein
T285_RS05655	1.67	6.38E-03	-	YggT family protein
T285_RS04545	1.67	1.28E-08	-	ABC transporter ATP-binding protein
T285_RS03700	1.67	1.54E-89	-	elongation factor Ts
T285_RS04495	1.66	1.09E-05	-	NUDIX hydrolase
T285_RS00670	1.64	3.61E-14	-	phosphate/phosphite/phosphonate ABC transporter substrate-binding protein
T285_RS08895	1.64	4.45E-02	-	LysR family transcriptional regulator
T285_RS09015	1.63	2.81E-09	-	type II toxin-antitoxin system mRNA interferase toxin%2C RelE/StbE family
T285_RS05720	1.63	2.48E-03	*mreD*	rod shape-determining protein MreD
T285_RS04140	1.62	6.20E-13	-	Nif3-like dinuclear metal center hexameric protein
T285_RS04055	1.62	3.05E-16	-	metal-sulfur cluster assembly factor
T285_RS00215	1.62	3.04E-03	-	DUF3923 family protein
T285_RS05275	1.61	4.79E-05	-	Tat (twin-arginine translocation) pathway signal sequence
T285_RS04080	1.60	1.04E-11	*ybeY*	rRNA maturation RNase YbeY

T285_RS06580	1.60	1.46E-54	-	glutamine synthetase
T285_RS04445	1.59	2.87E-23	-	alpha/beta hydrolase
T285_RS00125	1.58	1.75E-06	-	hypothetical protein
T285_RS03980	1.58	1.49E-04	-	cell division protein
T285_RS03885	1.55	1.43E-17	-	membrane protein
T285_RS00190	1.55	1.03E-27	-	alpha/beta hydrolase
T285_RS04550	1.55	4.38E-08	-	ABC transporter ATP-binding protein
T285_RS04040	1.55	1.80E-09	-	YitT family protein
T285_RS04665	1.54	6.44E-09	-	hypothetical protein
T285_RS04165	1.54	1.92E-04	-	hypothetical protein
T285_RS04020	1.53	5.31E-16	-	aminotransferase class I/II-fold pyridoxal phosphate-dependent enzyme
T285_RS04385	1.53	7.10E-03	-	hypothetical protein
T285_RS03720	1.52	7.49E-35	-	phosphatidate cytidylyltransferase
T285_RS04400	1.52	3.25E-05	-	GNAT family N-acetyltransferase
T285_RS03920	1.52	3.30E-20	-	methionine ABC transporter ATP-binding protein
T285_RS04250	1.51	9.60E-04	*pyrF*	orotidine-5'-phosphate decarboxylase
T285_RS04030	1.50	1.31E-33	-	SGNH/GDSL hydrolase family protein
T285_RS04075	1.50	4.57E-11	-	PhoH family protein
T285_RS00455	1.49	4.36E-04	-	amino acid ABC transporter ATP-binding protein
T285_RS04420	1.49	2.45E-03	-	hypothetical protein
T285_RS04085	1.49	8.59E-09	-	GTPase Era
T285_RS03370	1.48	3.82E-03	-	hypothetical protein
T285_RS03620	1.48	3.70E-28	*trmD*	tRNA (guanosine(37)-N1)-methyltransferase TrmD
T285_RS08140	1.47	5.19E-41	-	CvpA family protein
T285_RS08900	1.47	1.98E-13	-	hypothetical protein
T285_RS04830	1.46	1.91E-09	*tpx*	thiol peroxidase
T285_RS03725	1.45	7.43E-32	*rseP*	RIP metalloprotease RseP
T285_RS03710	1.45	1.30E-06	*frr*	ribosome recycling factor
T285_RS01180	1.45	1.06E-13	-	CTP synthase
T285_RS05940	1.45	1.10E-02	-	glycopeptide antibiotics resistance protein
T285_RS04360	1.44	1.43E-04	*eno*	phosphopyruvate hydratase
T285_RS03880	1.44	3.48E-14	*eno*	phosphopyruvate hydratase
T285_RS03615	1.44	3.69E-08	*rimM*	ribosome maturation factor RimM

T285_RS03415	1.43	3.07E-24	*nusB*	transcription antitermination factor NusB
T285_RS03715	1.42	3.57E-19	-	isoprenyl transferase
T285_RS04300	1.42	8.39E-07	-	glycosyltransferase
T285_RS05105	1.41	3.36E-16	-	YitT family protein
T285_RS06550	1.40	1.19E-11	-	hydroxymethylglutaryl-CoA reductase%2C degradative
T285_RS03730	1.40	2.23E-12	-	proline--tRNA ligase
T285_RS04375	1.39	2.28E-07	-	nitroreductase family protein
T285_RS08215	1.39	5.85E-28	-	alpha/beta fold hydrolase
T285_RS04705	1.39	2.05E-08	-	MarR family transcriptional regulator
T285_RS00825	1.38	1.46E-04	-	lysin
T285_RS04225	1.38	1.40E-03	-	heavy metal-binding domain-containing protein
T285_RS03445	1.38	8.90E-23	*recN*	DNA repair protein RecN
T285_RS01870	1.37	1.58E-18	-	MFS transporter
T285_RS04325	1.37	3.53E-03	-	nitroreductase family protein
T285_RS01135	1.36	3.97E-05	*pgmB*	beta-phosphoglucomutase
T285_RS07340	1.36	7.39E-03	-	hypothetical protein
T285_RS02210	1.36	3.75E-15	-	type II toxin-antitoxin system mRNA interferase toxin%2C RelE/StbE family
T285_RS03265	1.35	1.32E-12	-	cell division protein Fic
T285_RS07610	1.35	1.69E-19	-	TetR/AcrR family transcriptional regulator
T285_RS03760	1.34	5.69E-68	*infB*	translation initiation factor IF-2
T285_RS04850	1.34	9.72E-06	-	ATP-binding protein
T285_RS04800	1.34	1.23E-10	-	hypothetical protein
T285_RS04050	1.33	8.61E-13	*sdaAA*	L-serine ammonia-lyase%2C iron-sulfur-dependent%2C subunit alpha
T285_RS08730	1.33	6.18E-19	-	NCS2 family permease
T285_RS01325	1.33	1.74E-09	-	C69 family dipeptidase
T285_RS04160	1.33	1.61E-05	-	hypothetical protein
T285_RS03755	1.33	7.71E-09	-	50S ribosomal protein L7ae
T285_RS06180	1.33	2.66E-29	*eno*	phosphopyruvate hydratase
T285_RS03255	1.32	9.24E-38	*glmS*	glutamine--fructose-6-phosphate transaminase (isomerizing)
T285_RS04835	1.31	1.15E-04	-	antibiotic biosynthesis monooxygenase
T285_RS03960	1.31	3.35E-15	-	cell division protein
T285_RS03800	1.30	1.44E-15	*grpE*	nucleotide exchange factor GrpE

T285_RS05215	1.29	1.18E-39	-	bifunctional metallophosphatase/5'-nucleotidase
T285_RS05520	1.28	2.40E-04	-	30S ribosomal protein S20
T285_RS03990	1.27	1.64E-07	*prmA*	50S ribosomal protein L11 methyltransferase
T285_RS04435	1.26	3.96E-05	-	hypothetical protein
T285_RS06185	1.25	1.91E-21	-	triose-phosphate isomerase
T285_RS05685	1.24	1.22E-08	-	UDP-N-acetylmuramoyl-L-alanine--D-glutamate ligase
T285_RS06545	1.23	3.42E-26	-	hydroxymethylglutaryl-CoA synthase
T285_RS09030	1.23	5.57E-08	-	aldo/keto reductase
T285_RS04405	1.22	2.67E-03	-	hypothetical protein
T285_RS00185	1.22	4.88E-11	-	DEAD/DEAH box helicase
T285_RS07605	1.22	3.17E-07	-	MMPL family transporter
T285_RS03915	1.22	7.30E-04	-	MetQ/NlpA family ABC transporter substrate-binding protein
T285_RS02060	1.22	3.07E-24	-	GMP reductase
T285_RS03260	1.20	1.27E-18	-	helix-turn-helix domain-containing protein
T285_RS01355	1.20	4.89E-02	-	type B 50S ribosomal protein L31
T285_RS07620	1.20	3.13E-31	-	N-acetylglucosamine kinase
T285_RS04340	1.19	1.98E-16	-	SPFH domain-containing protein
T285_RS02205	1.19	5.57E-06	-	type II toxin-antitoxin system RelB/DinJ family antitoxin
T285_RS04070	1.19	5.98E-31	-	GatB/YqeY domain-containing protein
T285_RS03830	1.18	3.50E-03	-	adenine phosphoribosyltransferase
T285_RS03625	1.17	1.69E-02	*rplS*	50S ribosomal protein L19
T285_RS05860	1.17	8.34E-05	-	F0F1 ATP synthase subunit delta
T285_RS02490	1.17	2.24E-04	-	arginine--tRNA ligase
T285_RS04175	1.17	8.21E-04	*lepB*	signal peptidase I
T285_RS03750	1.17	1.43E-21	-	YlxR family protein
T285_RS03900	1.16	1.89E-02	-	helix-turn-helix transcriptional regulator
T285_RS03605	1.16	3.44E-10	*ffh*	signal recognition particle protein
T285_RS03170	1.16	2.83E-02	-	zinc ribbon domain-containing protein
T285_RS05705	1.16	2.63E-48	*rsmH*	16S rRNA (cytosine(1402)-N(4))-methyltransferase RsmH
T285_RS03945	1.15	2.65E-10	-	threonine/serine exporter family protein
T285_RS01925	1.15	1.27E-08	*rplL*	50S ribosomal protein L7/L12

T285_RS03695	1.14	2.35E-20	*rpsB*	30S ribosomal protein S2
T285_RS03765	1.14	5.37E-07	-	ribosome-binding factor A
T285_RS03405	1.14	1.45E-25	*efp*	elongation factor P
T285_RS05515	1.14	4.77E-02	*rpsO*	30S ribosomal protein S15
T285_RS03440	1.14	2.31E-23	-	TlyA family RNA methyltransferase
T285_RS04120	1.14	1.39E-04	-	amino acid ABC transporter permease
T285_RS06345	1.14	5.24E-05	*raiA*	ribosome-associated translation inhibitor RaiA
T285_RS02065	1.14	1.19E-04	-	adenylosuccinate synthase
T285_RS06485	1.13	3.97E-09	*ptsP*	phosphoenolpyruvate--protein phosphotransferase
T285_RS01140	1.13	1.01E-04	*ugpC*	sn-glycerol-3-phosphate ABC transporter ATP-binding protein UgpC
T285_RS03735	1.13	5.38E-14	-	PolC-type DNA polymerase III
T285_RS04805	1.12	3.54E-09	-	amidohydrolase family protein
T285_RS03795	1.12	4.49E-56	*hrcA*	heat-inducible transcriptional repressor HrcA
T285_RS04750	1.12	1.58E-03	-	LysR family transcriptional regulator
T285_RS00675	1.12	3.38E-20	*phnC*	phosphonate ABC transporter ATP-binding protein
T285_RS05680	1.11	3.55E-11	*murG*	undecaprenyldiphospho-muramoylpentapeptide beta-N-acetylglucosaminyltransferase
T285_RS03860	1.11	1.33E-33	-	HAD-IC family P-type ATPase
T285_RS03930	1.10	2.17E-09	-	class II fumarate hydratase
T285_RS03515	1.10	1.09E-04	-	thiamine diphosphokinase
T285_RS03015	1.10	2.73E-42	-	nucleoside hydrolase
T285_RS03770	1.09	2.93E-12	*truB*	tRNA pseudouridine(55) synthase TruB
T285_RS03435	1.09	1.27E-16	-	polyprenyl synthetase family protein
T285_RS05565	1.08	4.15E-24	*typA*	translational GTPase TypA
T285_RS04190	1.08	4.06E-03	-	hypothetical protein
T285_RS05040	1.08	1.35E-04	*hslU*	ATP-dependent protease ATPase subunit HslU
T285_RS02805	1.08	1.86E-07	-	amino acid ABC transporter permease
T285_RS00030	1.07	3.91E-23	-	30S ribosomal protein S6
T285_RS05045	1.07	9.22E-18	*hslV*	ATP-dependent protease subunit HslV
T285_RS04025	1.06	1.25E-04	*msrB*	peptide-methionine (R)-S-oxide reductase MsrB
T285_RS05700	1.06	3.65E-02	*ftsL*	cell division protein FtsL

T285_RS03165	1.06	2.59E-09	-	HAMP domain-containing histidine kinase
T285_RS01490	1.05	2.85E-36	*fusA*	elongation factor G
T285_RS09075	1.04	4.25E-03	*ssrA*	transfer-messenger RNA
T285_RS03745	1.04	3.02E-32	*nusA*	transcription termination/antitermination protein NusA
T285_RS08785	1.03	4.60E-16	-	aluminum resistance protein
T285_RS05710	1.02	2.45E-08	*mraZ*	division/cell wall cluster transcriptional repressor MraZ
T285_RS07720	1.02	6.44E-19	-	nicotinamide mononucleotide transporter
T285_RS06190	1.02	2.19E-10	-	phosphoglycerate kinase
T285_RS03420	1.01	7.29E-09	-	bifunctional methylenetetrahydrofolate dehydrogenase/methenyltetrahydrofolate cyclohydrolase
T285_RS03775	1.01	5.36E-22	*ribF*	riboflavin biosynthesis protein RibF
T285_RS05850	1.01	3.51E-18	-	F0F1 ATP synthase subunit gamma
T285_RS05035	1.01	2.11E-23	-	aldose 1-epimerase family protein
T285_RS03610	1.01	6.94E-10	*rpsP*	30S ribosomal protein S16
T285_RS05500	1.01	1.26E-07	*tuf*	elongation factor Tu
T285_RS03935	1.00	4.70E-06	-	flavocytochrome c
T285_RS06570	1.00	1.77E-03	-	HAD family hydrolase

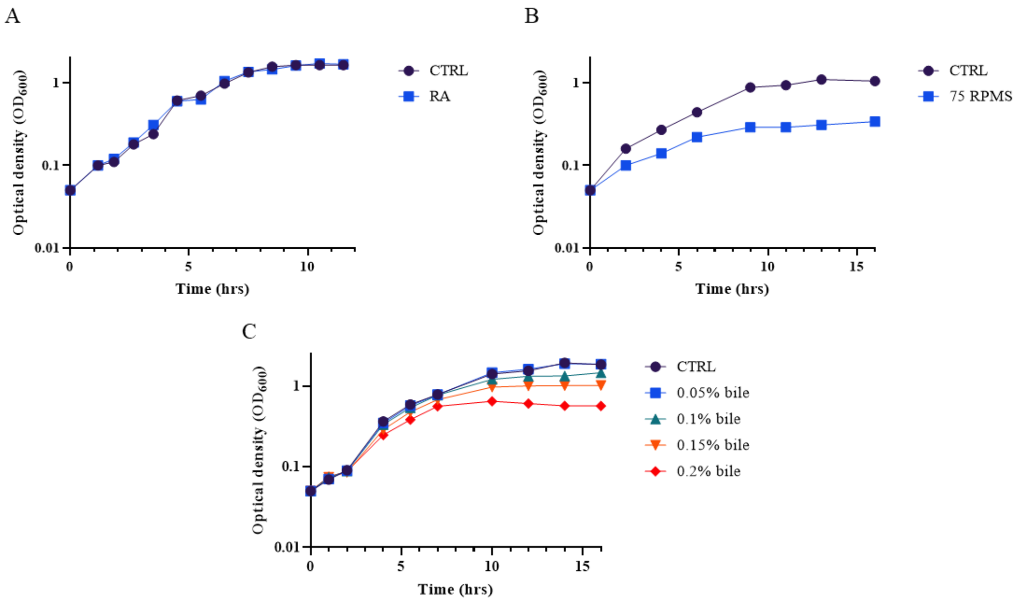

Supplementary Figure 1: Growth curves of *L. johnsonii* N6.2 grown under static conditions in vdMRS (CTRL) and with the following modifications: **A.** 100 µM rosmarinic acid (RA), **B.** shaking at 75 rpms, **C.** 0.05 % - 0.2 % bovine bile. Growth curves were performed in biological triplicates, data points show the mean of the three biological replicates, and error bars represent standard deviation.

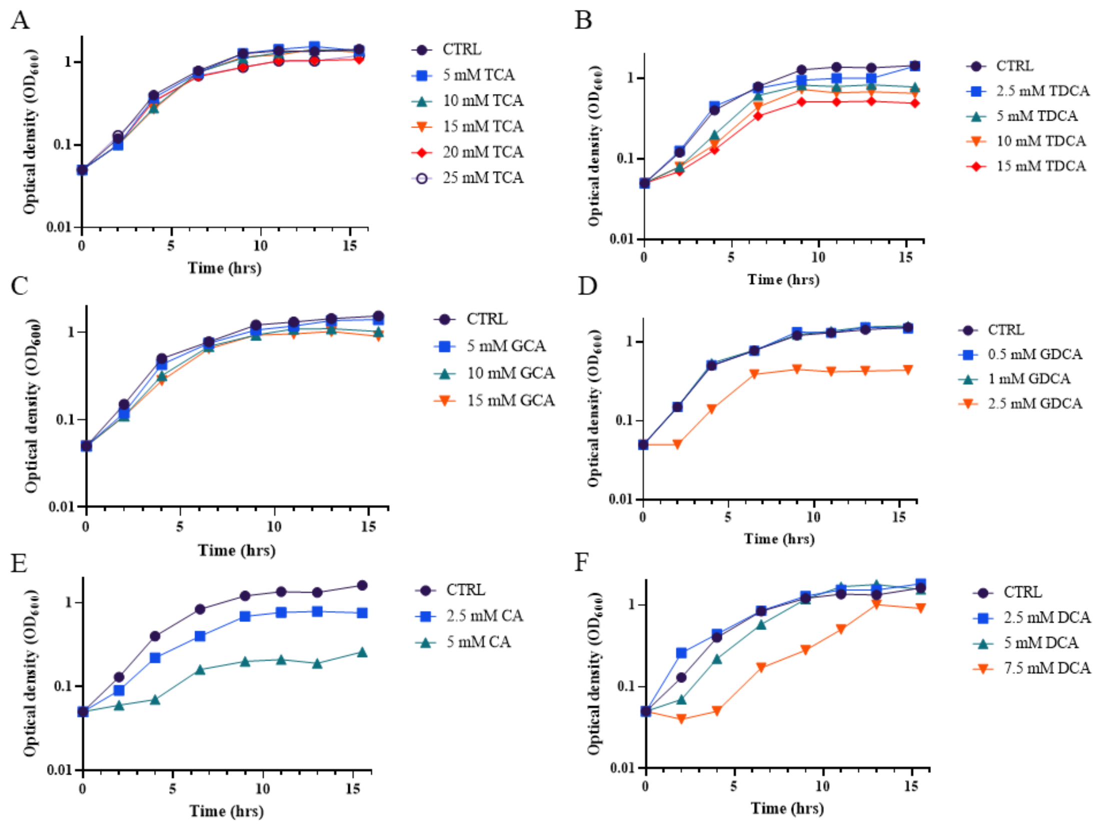

Supplementary Figure 2: Growth curves of *L. johnsonii* N6.2 grown under static conditions in vdMRS (CTRL) and with the following modifications: **A.** 5 – 25 mM taurocholic acid (TCA), **B.** 2.5 – 15 mM taurodeoxycholic acid (TDCA), **C.** 5 – 15 mM glycocholic acid (GCA), **D.** 0.5 – 2.5 mM glycodeoxycholic acid (GDCA), **E.** 2.5 and 5 mM cholic acid (CA), **F.** 2.5 – 7.5 mM deoxycholic acid (DCA). Growth curves were performed in biological triplicates, data points show the mean of the three biological replicates, and error bars represent standard deviation.

A

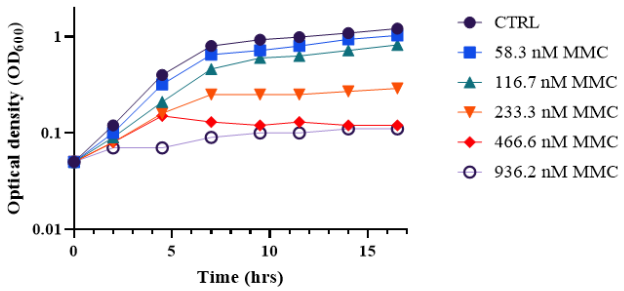

Supplementary Figure 3: Growth curves of *L. johnsonii* N6.2 grown under static conditions in vdMRS (CTRL) and with 58.3 – 936.2 nM mitomycin C (MMC). Growth curves were performed in biological triplicates, data points show the mean of the three biological replicates, and error bars represent standard deviation.

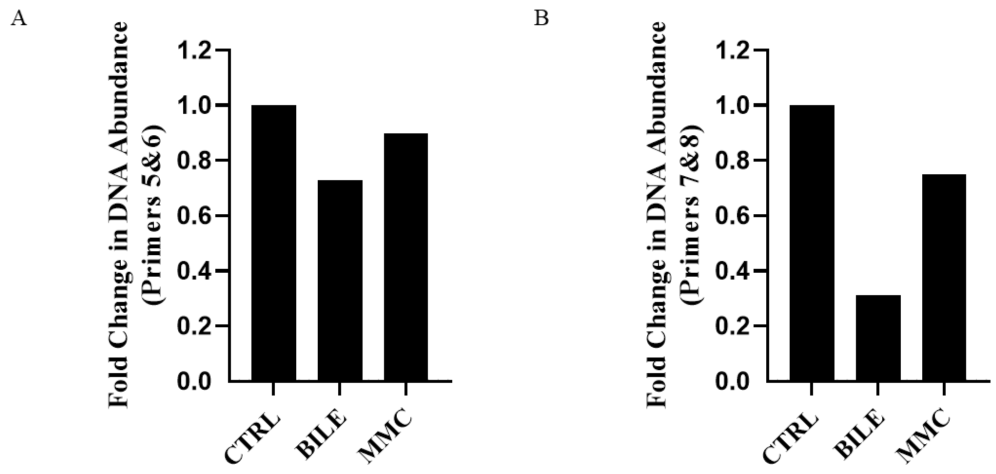

Supplementary Figure 4: qPCR using DNA extracted from *L. johnsonii* N6.2 grown in the control, bile, or mitomycin C conditions to test abundance of DNA within the incomplete prophage regions normalized to DNA outside of this region. **A.** qPCR of P1 DNA normalized to amplification with primers 9 + 10. **B.** qPCR of DNA directly downstream of P1 normalized to amplification with primers 9 + 10.

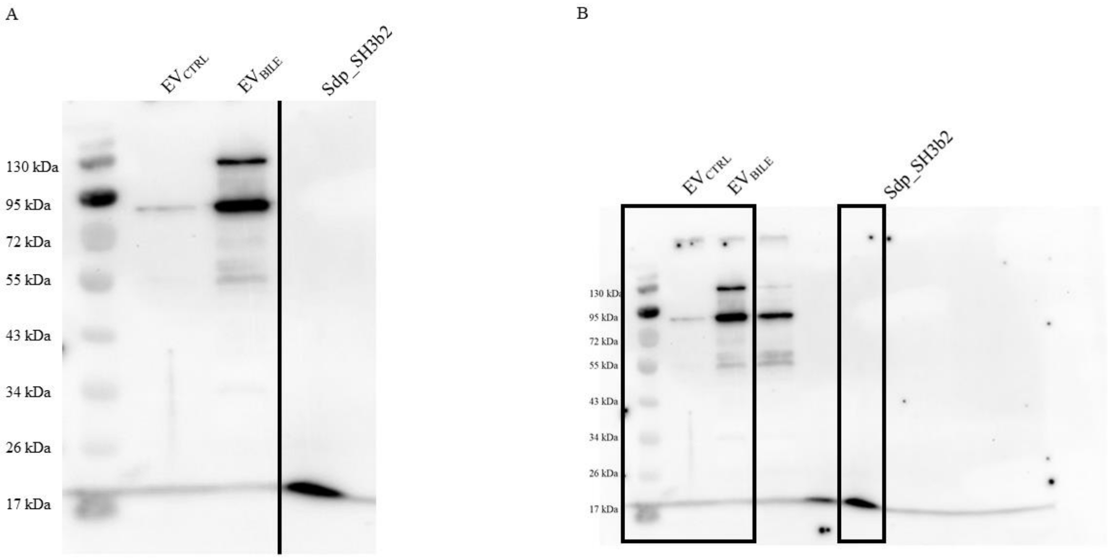

Supplementary Figure 5: Differential abundance of Sdp in EV_{CTRL} vs EV_{BILE}. **A.** Western blot performed using anti-Sdp_SH3b2 with 2.2 x 10^9 EVs per well or 10 ng purified Sdp_SH3b2 protein as a positive control. The purified Sdp_SH3b2 domain has an expected size of ~15 kDa, whereas the whole Sdp protein present in the EVs has an expected size of ~100 kDa. **B.** Uncropped blot from A.
